# Supplementary material for: Controlling an altermagnetic spin density wave in the kagome magnet CsCr3Sb5
Source: Nat Commun. 2026 Jun 1;17:7041. doi: 10.1038/s41467-026-73976-3 (PMC13392461; doi:10.1038/s41467-026-73976-3)
Supplement: Supplementary file 1 — Supplementary Information [file 41467_2026_73976_MOESM1_ESM.pdf]

Supplementary Materials

Controlling an altermagnetic spin density wave in the kagome magnet $CsCr_3Sb_5$

Zihao Huang[1,#], Chenchao Xu[2,3,#], Yande Que[1,#], Yi Liu[3,4,#], Yunhao Wang[5], Shiyu Zhu[5], Ranjith Shivajirao[1], Zheng Jue Tong[1], Amit Kumar[1], Chao Cao[3,*], Guang-Han Cao[3,*], Hong-Jun Gao[5], Bent Weber[1,*]

[1] *School of Physical and Mathematical Sciences, Nanyang Technological University, Singapore 637371, Singapore*

[2] *School of Physics, Hangzhou Normal University, Hangzhou 310036, P. R. China.*

[3] *School of Physics, Zhejiang University, Hangzhou 310027, PR China.*

[4] *Department of Applied Physics, Key Laboratory of Quantum Precision Measurement of Zhejiang Province, Zhejiang University of Technology, Hangzhou, PR China.*

[5] *Beijing National Center for Condensed Matter Physics and Institute of Physics, Chinese Academy of Sciences, Beijing 100190, PR China*

[#]These authors contributed equally to this work

[*]Correspondence author, Email address: *b.weber@ntu.edu.sg; ghcao@zju.edu.cn*; *ccao@zju.edu.cn*

This file includes:

Sections A-G

Figs. S1-S23

Table 1

Section A. Spin dynamics simulation of the SDW under the magnetic field

In correlated electron systems where CDW and SDW instabilities coexist or compete, an external magnetic field can couple directly to the spin through the Zeeman effect, tuning the SDW order. In $CsCr_3Sb_5$, the CDW order is strongly coupled to the magnetic pattern as shown in our results, as well as recent works[1,2].

Consequently, we expect that a magnetic-field-manipulated spin order shall induce a corresponding CDW manipulation. From our additional theoretical analysis (below), we conclude that the magnetic-field-induced CDW manipulation is therefore mediated through the corresponding manipulation of the underlying SDW phase. The associated spin dynamics in a magnetic field can be qualitatively understood in analogy to a simple toy model based on a one-dimensional antiferromagnetic spin chain in a magnetic field.

We consider the antiferromagnetic Heisenberg spin-exchange Hamiltonian,

$$\mathcal{H} = -J\sum_{\langle ij\rangle}\mathbf{S}_i \cdot \mathbf{S}_j - B_z\sum_i S_i^z,$$

in a magnetic field B_z (lattice sites L=200) with periodic boundary conditions.

To describe the dynamics of the chain, we use the Landau-Lifshitz-Gilbert (LLG) equation,

$$\frac{d\mathbf{S}}{dt} = -\gamma\mathbf{S} \times \mathrm{H}_{\mathrm{eff}} + \alpha\mathbf{S} \times \frac{d\mathbf{S}}{dt} \;,$$

with an effective field $\mathbf{H}_{\mathrm{eff},i} = -\frac{\partial\mathcal{H}}{\partial\mathbf{S}_i}$ and Gilbert damping parameter $\alpha = 0.01$.

We further assume an in-plane antiferromagnetic ground state as the initial configuration in our LLG simulations. This is justified by our DFT calculations, which show that the in-plane magnetic moment configuration is lower in energy than the out-of-plane (c-axis) configuration by about 2 meV/f.u.. Within the plane, the energy difference between different orientations is negligible (<1 meV/f.u.).

The simulation results are present in Fig. S15 after the system reaches a steady state. An out-of-plane magnetic field not only induces precession of in-plane spins (Fig. S15**a**), but also introduces a weak ferromagnetic moment along the z-direction (Figs. S15**b**), driving a transition from a collinear AFM state to a canted AFM phase.

Importantly, the spatial distribution of S_x, shown in Fig. S15**c**, exhibits a cosine-wave modulation. Similar to what is observed in the experiments, the phase of this in-plane SDW exhibits a linear dependence on the magnetic field (Fig. S15**d**). This trend qualitatively aligns with the linear variation of the CDW stripe phase observed experimentally (Fig. 5**d** of the main text).

Based on this simple toy model and our experimental results, we can obtain a rough estimate of the exchange interaction *J*. As observed in the experiment, the rate of phase shift in magnetic field is approximately $\pi/10$ T^{-1} (Fig. 5**d**). The calculations yield $\pi/(0.05J)$ (Fig. S15**d**), giving $|J|\sim$ 20 meV, taking the corresponding Zeeman energy (~0.1 meV at 1 T) into account. This estimate is consistent with the anisotropic nearest-neighbor exchange interaction *J* (ranging from -10meV to -40 meV) calculated for the altermagnetic SDW ground state of $CsCr_3Sb_5$, using the tb2J package[3]. Such near-quantitative agreement is remarkable, given the simplicity of the 1D toy model in describing the SDW/CDW sliding mechanism.

Section B. Identification of the CDW gap

The CDW gap feature can be revealed in high-resolution spectroscopy within a narrow bias window, as shown in Fig. 1**g**. In this measurement (V_b=−50 mV, I=2 nA, $V_{lock\text{-}in}$ =0.5 mV), the energy resolution is well below 1 meV, allowing us to clearly observe a pair of pronounced spectral kinks symmetrically located at approximately ±9 meV, which we attribute to the opening of a CDW gap.

The magnitude of this gap is consistent with the transport measurements. Previous studies report a density wave transition in $CsCr_3Sb_5$ at $T_{\mathrm{DW}} \approx 55\mathrm{K}$[4]. Using the mean-field estimate $\Delta \approx 1.76 k_{\mathrm{B}} T_{\mathrm{DW}}$, one obtains $\Delta \approx 8.4\,\mathrm{meV}$, which is in excellent agreement with the gap magnitude observed in our measurements.

To further substantiate the identification of the CDW gap, we have added a $\mathrm{d}I/\mathrm{d}V$ linecut analysis illustrating the spatial evolution of the gap across the CDW modulation (Fig. S16). As shown by line cuts taken both perpendicular to the stripes (Fig. S16**b**) and along the stripe direction (Fig. S16**c**), the gap magnitude exhibits clear spatial modulations that track the CDW pattern. This direct correlation between the local gap strength and the CDW modulation provides further compelling evidence that the observed spectral kinks originate from the CDW order.

In addition, one may expect multiple associated energy scales in the tunneling spectra, given the presence of two structural components in the CDW. In Fig. 1**g**, the most prominent spectral feature is a pair of pronounced kinks at approximately ±9 meV, which we attribute to the primary CDW gap. We have performed additional analysis and found a second, weaker kink structure located within the larger gap.

Our analysis is shown in Fig. S21 for two separate data sets, the original Fig. 1**g** as well as a new data set. In both, we carried out a second-derivative analysis. This analysis reveals two distinct energy scales: a dominant pair of peaks corresponding to the ±9 meV gap, as well as a secondary pair of features around ±3 meV. The latter may be associated with the second CDW structural component.

Section C. Field of view alignment procedure

The circular markers shown in Fig. 3 denote the same three atomic-scale defects, which serve as fixed reference points to precisely align the imaging area across different magnetic fields. These defects are field-insensitive and remain unchanged throughout the measurements, allowing us to reliably track the same region of the surface with picometer precision.

To further ensure that the observed CDW phase shifts are intrinsic and not caused by instrumental drift or image misregistration, we had implemented the following procedures at each magnetic field:

1. **Continuous tip engagement:** The STM tip is not withdrawn during magnetic field ramping, which significantly suppresses piezo-related drift associated with tip retraction and re-approach.
2. **Post-field stabilization:** After reaching the target magnetic field, we allow sufficient time for the STM to thermally and mechanically stabilize before imaging.
3. **High-bias reference imaging:** We first acquire a relatively large field-of-view topograph at high bias (Fig. S17**a**), where the CDW contrast is weak allowing atomic defects to be clearly identified. These high-bias images, together with the defect markers, serve as an internal reference to confirm spatial alignment across fields.
4. **Precise field-of-view registration:** Guided by the defect positions in the high-bias images, we then reduce the scan area and carefully align the field of view (Fig. S17**b**) such that its boundaries are fixed relative to the three reference defects (D1–D3), ensuring consistent positioning for all low-bias scans (Fig. S17**c**).

These procedures include a comparison of high-bias and low-bias data (Figs. S17**b** and S17**c**), confirming precise alignment for each applied magnetic field, and any residual drift is negligible compared to the observed CDW phase shifts. We can estimate the alignment uncertainty by comparing the position of defect D1 at different magnetic fields, as demonstrated in Figs. S17**d**-**f**. As shown, the positions of D1 at 0 T and 9 T exhibit negligible mismatch away from the left scan boundary (Fig. S17**d**). Gaussian fits yield an estimated alignment uncertainty of approximately 26 pm (Figs. S17**e**-**f**). Given that $4a_0$~2200 pm, the relative uncertainty is about 1%.

We further note that, as shown in the Fig. S8, the CDW stripes undergo pronounced field-induced reconfiguration while the reference defects remain spatially fixed. This

directly demonstrates that the observed phase evolution originates from genuine CDW sliding rather than instrumental drift or scan misregistration.

Section D. Calibration of the effective electron temperature

The effective electron temperature (T_{eff}) of the scanning tunneling microscope (STM) in our dilution refrigerator was calibrated in situ using spectroscopic measurements on a superconducting aluminum (Al) bulk polycrystal. Prior to the spectroscopic measurement, the Al polycrystal surface was cleaned through a few cycles of argon ions sputtering followed by annealing at ~350 ˚C. Following this preparation, the sample was transferred directly into the STM head at UHV conditions without exposure to ambient atmosphere. The measured density of states is influenced by thermal broadening, characterized by T_{eff}. To extract this parameter, the data were fitted using the Maki-BCS model for superconductivity in the presence of pair-breaking effects[5-9]. Within this framework, the normalized superconducting density of states $N_s(E)$ is given by:

$$\rho(E) = Re\left(\frac{u}{\sqrt{u^2 - 1}}\right)$$

$$u = \frac{E}{\Delta} + \zeta \frac{u}{\sqrt{1 - u^2}}$$

Where Δ is the superconducting energy gap and ζ is the pair-breaking parameter. To account for thermal broadening, this intrinsic density of states is convolved with the derivative of the Fermi-Dirac distribution function. The fitting procedure, with Δ, ζ, and T_{eff} as free parameters, yielded an effective temperature of $T_{\mathrm{eff}} = (155.7 \pm 2.3)$ mK. A representative spectrum and the corresponding best-fit curve are shown in Supplementary Figure 18. This T_{eff} corresponds to an energy resolution ($\delta E \approx 3.5 k_B T_{\mathrm{eff}}$) of ~46 μeV, which is significantly smaller than the characteristic energy scales (~9 meV) of the charge density wave phenomena investigated in this work, confirming the adequacy of our instrumental resolution.

Section E. Criteria for determining domain boundaries

We identify the domain boundaries directly from atomic-resolution STM topography in Fig. 4**a**. Specifically, the boundaries are determined based on the following criteria (Fig. S20):

1. **Abrupt changes in stripe intensity within individual CDW stripes**, signaling a transition between internal structures of CDW pattern (Fig. S20**a**).
2. **Stripe splitting or merging events**, which occur also between internal structures of CDW pattern (Fig. S20**b**).
3. **Direct transitions between $4a_0$ and $8a_0$ stripe periodicities**, clearly visible in real space (Fig. S20**c**).

These criteria enable identification of atomically sharp boundaries and are overall consistent with the variations of CDW amplitude and phase obtained from the single-q lock-in analysis, while additionally revealing finer atomic-scale details.

Section F. Magnetic force microscopy measurement

MFM is primarily sensitive to stray magnetic fields arising from a net magnetization and is therefore well suited to detecting ferromagnetic or ferrimagnetic domain structures[10, 11]. In contrast, the SDW identified in $CsCr_3Sb_5$ is altermagnetic in nature without any net magnetization and the magnetic-field-induced CDW sliding does not involve any magnetic phase transition. Over a wide range of applied magnetic fields, $CsCr_3Sb_5$ is expected to retain its altermagnetic character, exhibiting no net magnetization and generating no magnetic domains associated with other magnetic phases. Consequently, MFM is intrinsically insensitive to this form of magnetic order.

We have performed low-temperature MFM measurements down to 50 mK under varying magnetic fields. The MFM experiments were captured by a commercial magnetic force microscope (attoAFM, attocube) using a commercial magnetic tip (MFMR, Nanoword) based on a closed-cycle He cryostat (Bluefors) and dilution refrigerator system (Bluefors), combined with a vector magnet (9-3-1, Bluefors). The scanning probe system was operated at the resonance frequency of the magnetic tip, approximately 70 kHz. The MFM signal, i.e., the resonance frequency shift (Δf), is proportional to the out-of-plane stray field gradient. As shown in Fig. S22, no discernible magnetic contrast or field-dependent evolution is observed on the micrometer scale. The small blue spot is likely a defective region of the crystal serving as a reference point for scanning the same field of view. The absence of MFM signal is fully consistent with the altermagnetic nature of the SDW ground state inferred from our STM observations and DFT calculations, and further excludes the magnetic phase transition as the origin of the magnetic-field-induced sliding CDW.

Section G. Inspection of piezo-magnetic response

A piezomagnetic effect can manifest as a magnetic-field-induced lattice deformation. Experimentally, this mechanism is supported by a measurable change in the lattice constants, visible as a systematic shift in the Bragg peak positions in Fourier-transformed STM images under different magnetic fields.

If the magnetic-field-induced CDW sliding observed in our experiment originated from a piezomagnetic response, a similar field-dependent modification of the lattice constant would be expected. Specifically, the magnitudes of the Bragg wave vectors should change with applied magnetic field.

To directly test this possibility, we carefully compared the Bragg peak positions extracted from STM images acquired at 0 T, 4.5 T, and 9 T. The results are presented in Fig. S23. As shown in Fig. S23**c**, the Bragg peaks at different fields overlap within experimental uncertainty, indicating that the lattice constants remain unchanged across the entire magnetic-field range.

This observation demonstrates that any piezomagnetic lattice distortion, if present, is negligible in our system. Consequently, the magnetic-field-induced CDW evolution we report cannot be explained by a piezomagnetic mechanism.

Figures

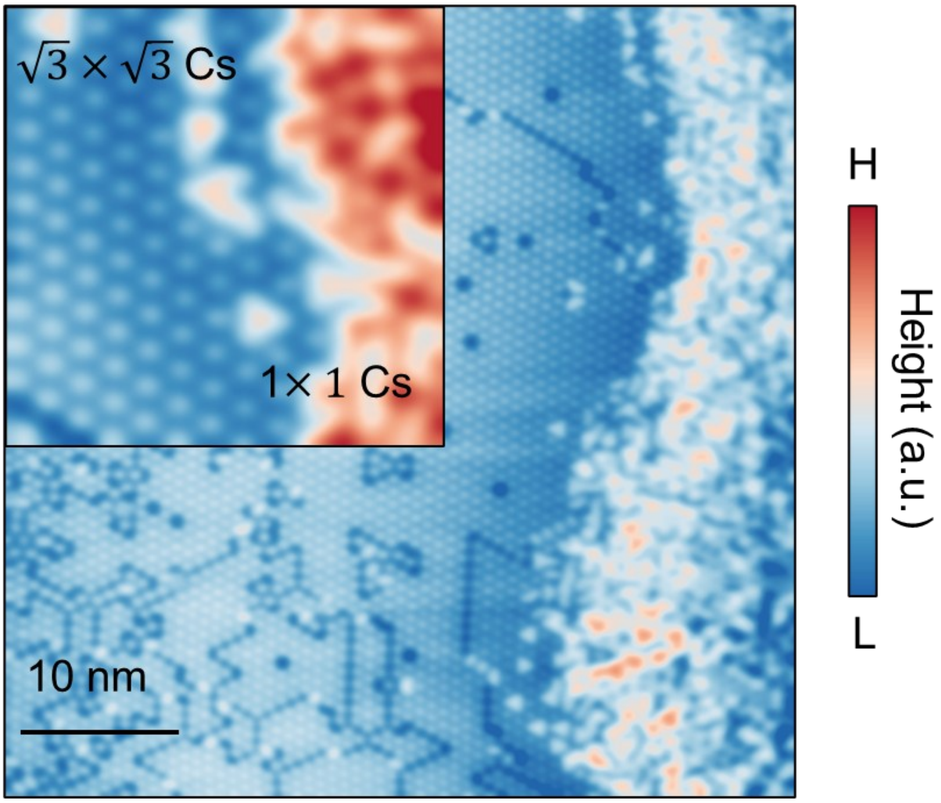

Fig. S1. Cs-terminated surface. STM topographic image of Cs-terminated surface. Inset shows 1×1 Cs surface and $\sqrt{3}\times\sqrt{3}$ reconstruction Cs surface. Measurement conditions : V_b=-800 mV, I=1 nA.

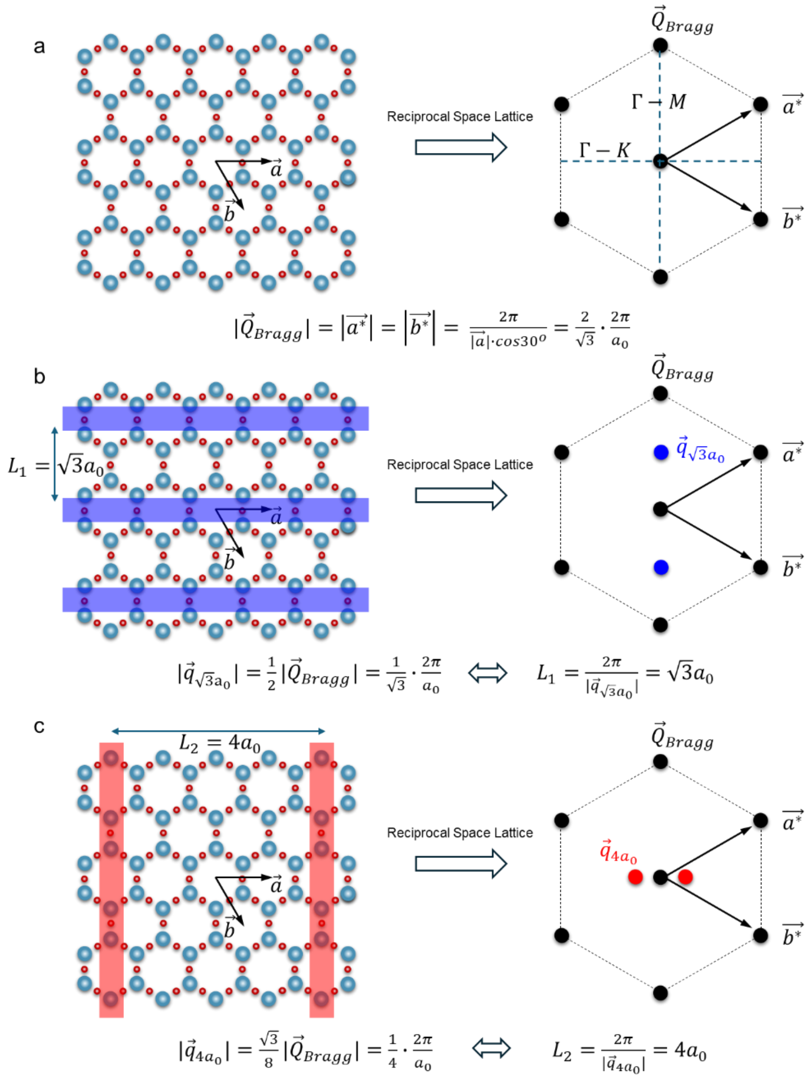

Fig. S2. Schematic illustrations of CDW stripes and their scattering vectors. **a**, Schematic of the hexagonal atomic lattice and its reciprocal lattice. The magnitude of the Bragg vector is given by: $|\vec{Q}_{Bragg}| = |\vec{a^*}| = |\vec{b^*}| = \frac{2\pi}{|\vec{a}| \cdot cos30^o} = \frac{2}{\sqrt{3}} \cdot \frac{2\pi}{a_0}$. **b**, Schematic of a CDW stripe with a period of $\sqrt{3}a_0$ and its corresponding scattering vector $\vec{q}_{\sqrt{3}a_0}$, which aligns along the $\Gamma - M$ direction. Its magnitude is: $|\vec{q}_{\sqrt{3}a_0}| = \frac{1}{2}|\vec{Q}_{Bragg}| = \frac{1}{\sqrt{3}} \cdot \frac{2\pi}{a_0}$. c, Schematic of a CDW stripe with a period of $4a_0$ and its corresponding scattering vector $\vec{q}_{4a_0}$, which aligns along the $\Gamma - K$ directionIts. Its magnitude is: $|\vec{q}_{4a_0}| = \frac{\sqrt{3}}{8}|\vec{Q}_{Bragg}| = \frac{1}{4} \cdot \frac{2\pi}{a_0}$. The scattering vectors are labeled according to their real-space periodicity.

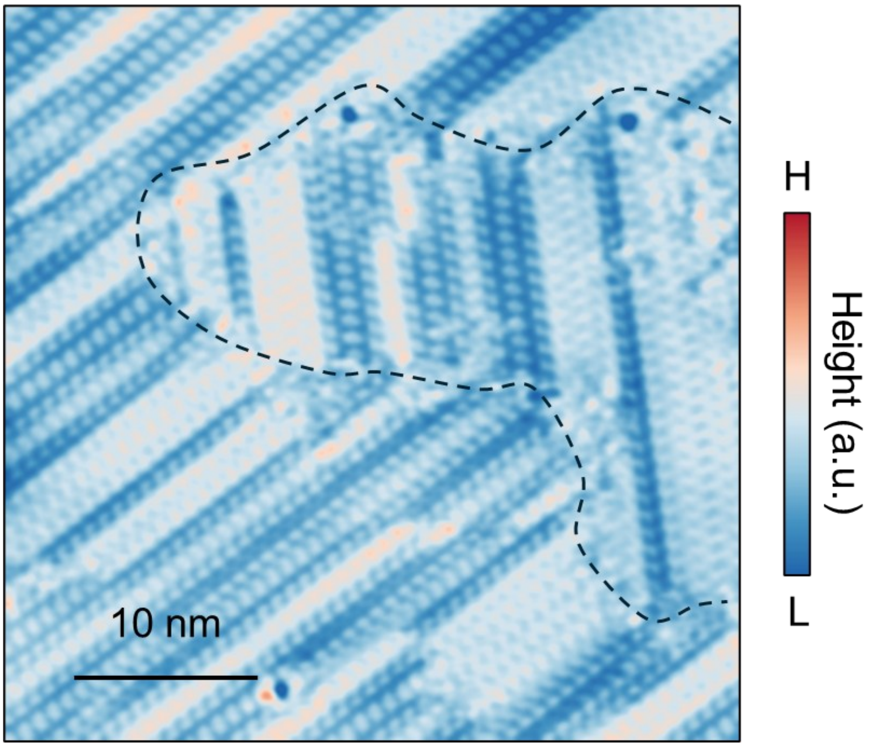

Fig. S3. Coexistence of CDW domains with distinct orientations. STM topographic image of a region shows domains where the CDW stripes are oriented in different directions. Measurement conditions : V_b=-50 mV, I=1 nA.

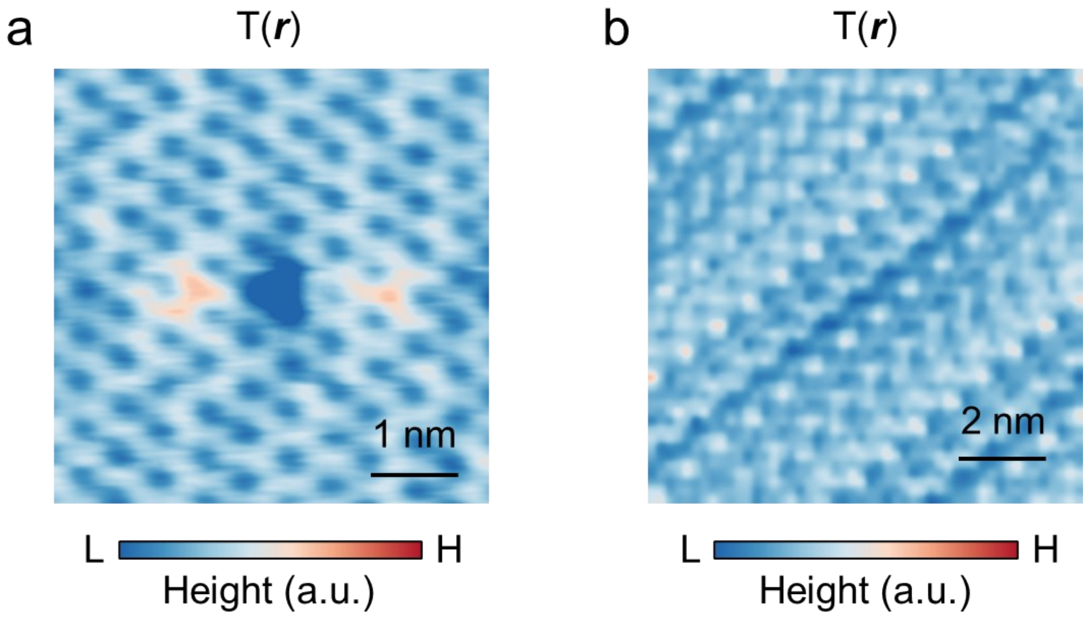

Fig. S4. The region for Inversion contrast measurements. **a**, STM topographic image of the Sb2 surface with $4a_0 \times \sqrt{3}a_0$ CDW of Fig. 2**g**. Measurement conditions: V_b=-900 mV, I=1 nA. **b**, STM topographic image of the Sb2 surface with $8a_0 \times \sqrt{3}a_0$ CDW of Fig. 2**h**. Measurement conditions: V_b=90 mV, I=1 nA.

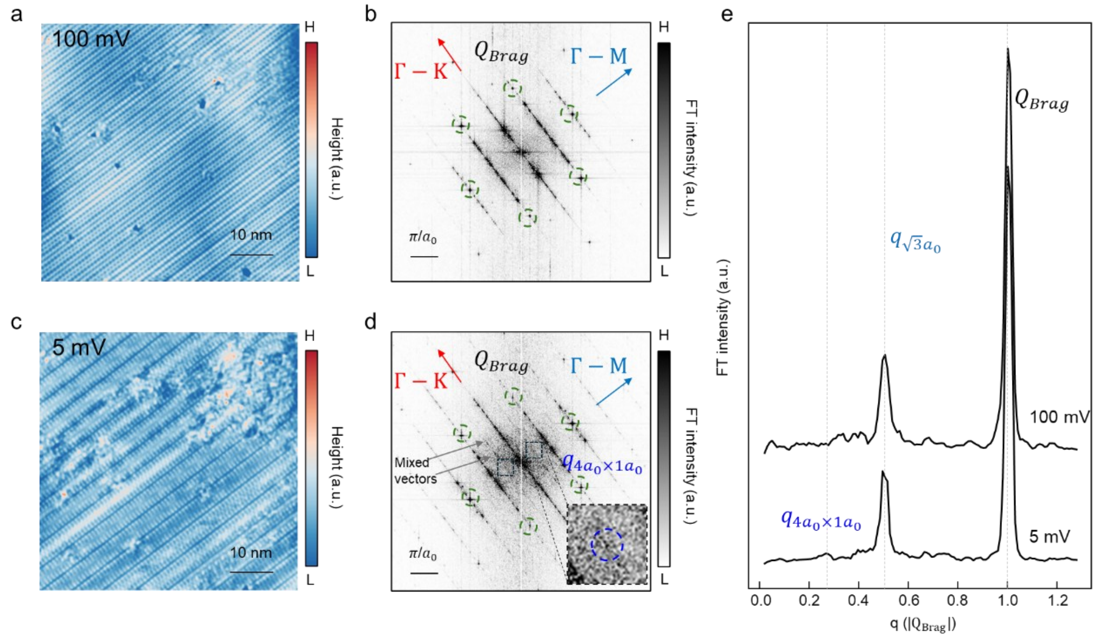

Fig. S5. Signatures of the bulk $4a_0 \times 1a_0$ CDW. a, STM topographic image taken at 100 mV with large tip-sample distance. Measurement conditions : V_b=100 mV, I=2 nA. **b**, FT of (a), showing no scattering vector at the $1/4|Q_{Brag}|$ position along the $\Gamma - M$ direction. **c**, STM topographic image taken at 5 mV with small tip-sample distance. Measurement conditions : V_b=5 mV, I=0.3 nA. **d**, FT of (c), revealing a faint scattering vector at the $1/4|Q_{Brag}|$ position along $\Gamma - M$, along with corresponding mixed vectors with q_{8a_0} along the $\Gamma - K$ direction. **e**, Linecut comparison along the $\Gamma - M$ direction between high tip-sample distance (100 mV) and low tip-sample distance (5 mV), illustrating the emergence of a weak scattering peak at low tip–sample distance.

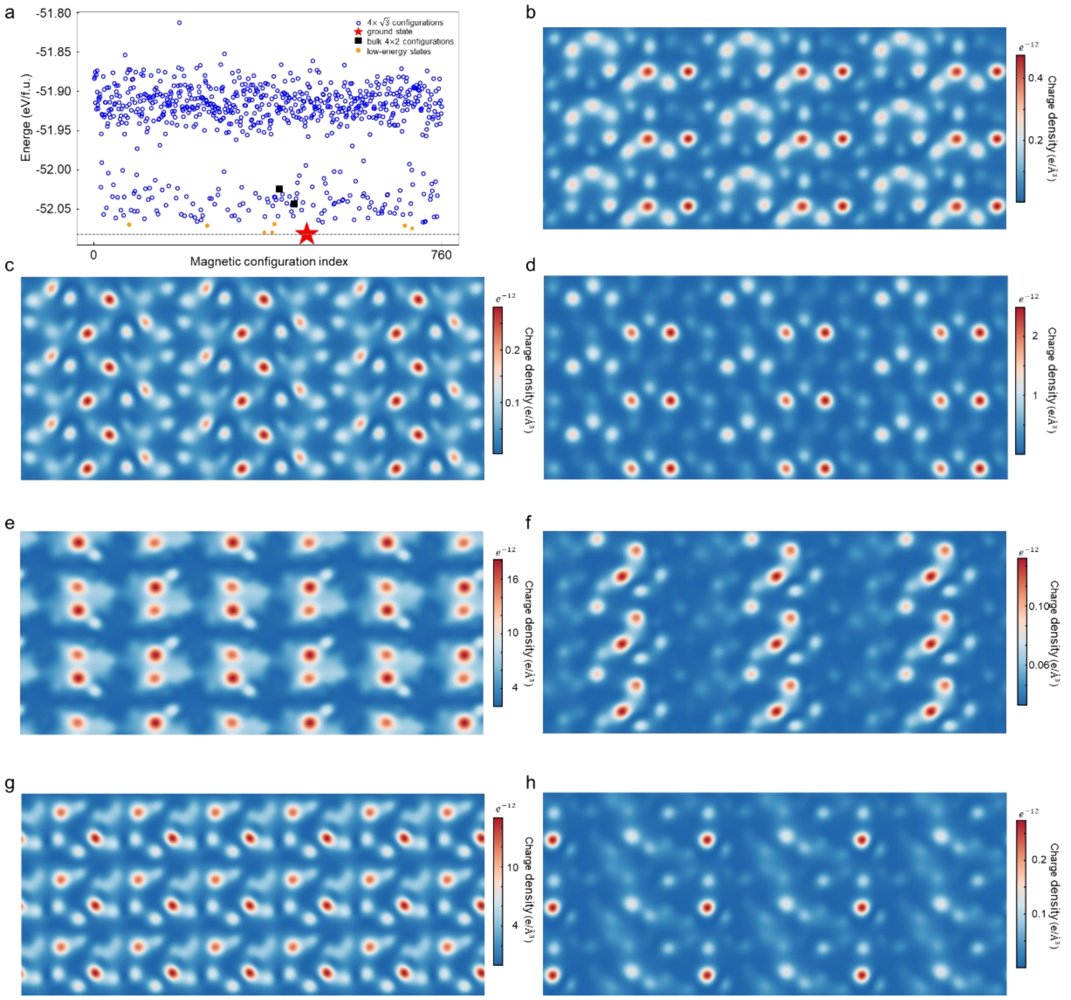

Fig. S6. Energy comparison of SDW configurations and calculated charge density modulations for low-energy SDW configurations. a, DFT-calculated total energies of different SDW configurations. For the surface monolayer, the $4a_0 \times \sqrt{3}a_0$ SDW ground state is energetically favored over the bulk $4a_0 \times 2a_0$ SDW. The energy gap between the ground state and the next-lowest-energy state corresponds to 18 K per $CsCr_3Sb_5$ unit cell, significantly exceeding the measurement temperature of 50 mK. **b-h**, Simulated charge density patterns for seven low-energy SDW configurations that are energetically, which lie slightly above the ground-state configuration shown in Fig. 3. None of these configurations reproduce the charge modulations observed in the STM topographic images.

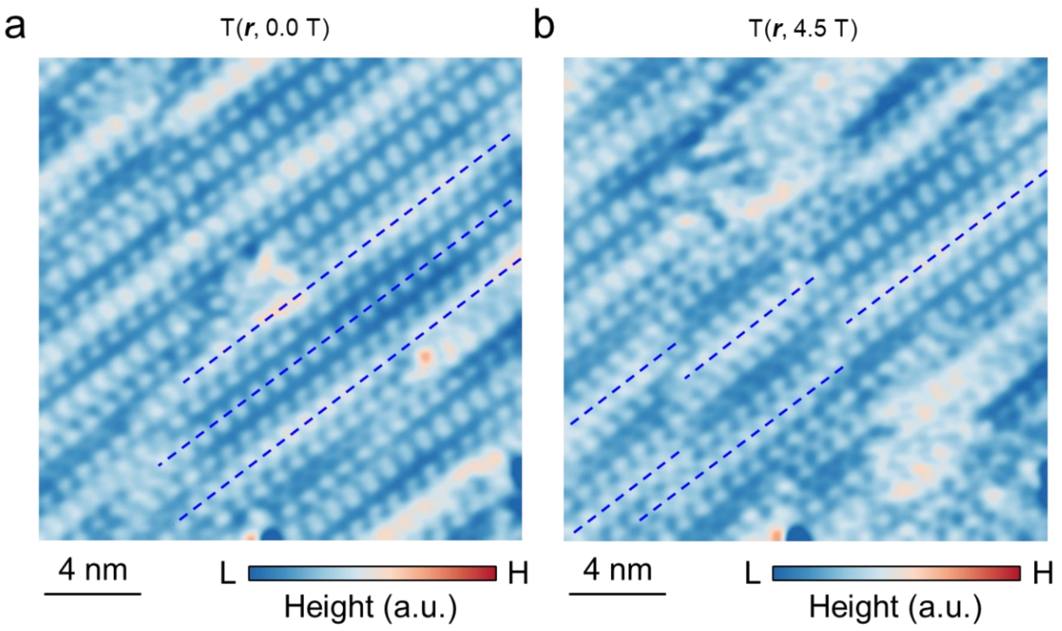

Fig. S7. CDW stripe splitting. **a**, STM topographic images T(***r***, ***B***$_z$) of mixed $4a_0 \times \sqrt{3}a_0$ ad $8a_0 \times \sqrt{3}a_0$ CDW region at 0 T. Measurement conditions: V_b=-50 mV, I=1 nA. **b**, STM topographic images T(***r***, ***B***$_z$) of the same region at 4.5 T. Measurement conditions: V_b=-50 mV, I=1 nA. The dotted lines mark the stripe positions, revealing stripe splitting.

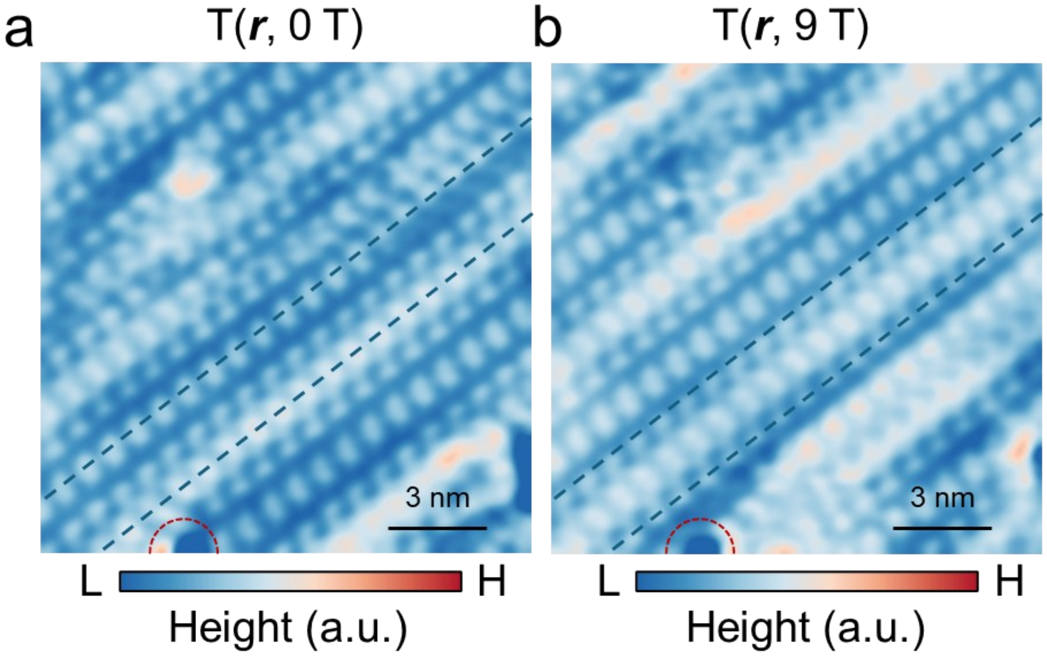

Fig. S8. Magnetic field-induced stripe shift. **a**, STM topographic images T(***r***, ***B****z*) of mixed $4a_0 \times \sqrt{3}a_0$ ad $8a_0 \times \sqrt{3}a_0$ CDW region at 0 T. Measurement conditions: V_b=-50 mV, I=1 nA. **b**, STM topographic images T(***r***, ***B****z*) of the same region at 9 T. Measurement conditions: V_b=-50 mV, I=1 nA. The dotted lines mark the stripe positions, showing a π phase shift of the stripe.

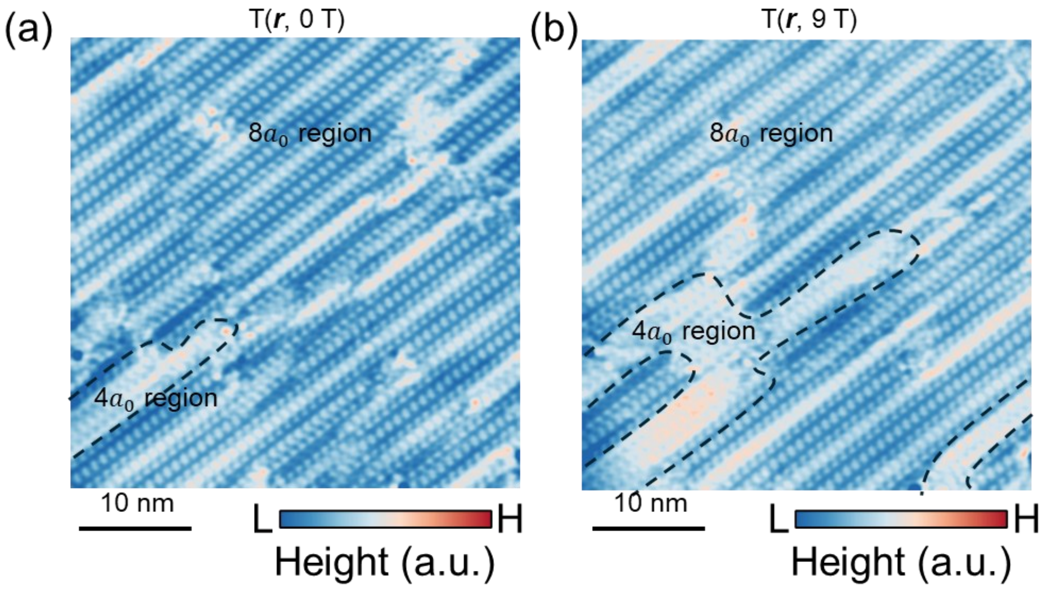

Fig. S9. interconversion between $4\boldsymbol{a_0} \times \sqrt{3}\boldsymbol{a_0}$ ad $8\boldsymbol{a_0} \times \sqrt{3}\boldsymbol{a_0}$ CDW. **a**, STM topographic images T(***r***, ***B****z*) at 0 T. Measurement conditions: V_b=-50 mV, I=1 nA. **b**, STM topographic images T(***r***, ***B****z*) at 9 T. Measurement conditions: V_b=-50 mV, I=1 nA. The dotted lines mark the domain walls between $4a_0 \times \sqrt{3}a_0$ ad $8a_0 \times \sqrt{3}a_0$ CDW region.

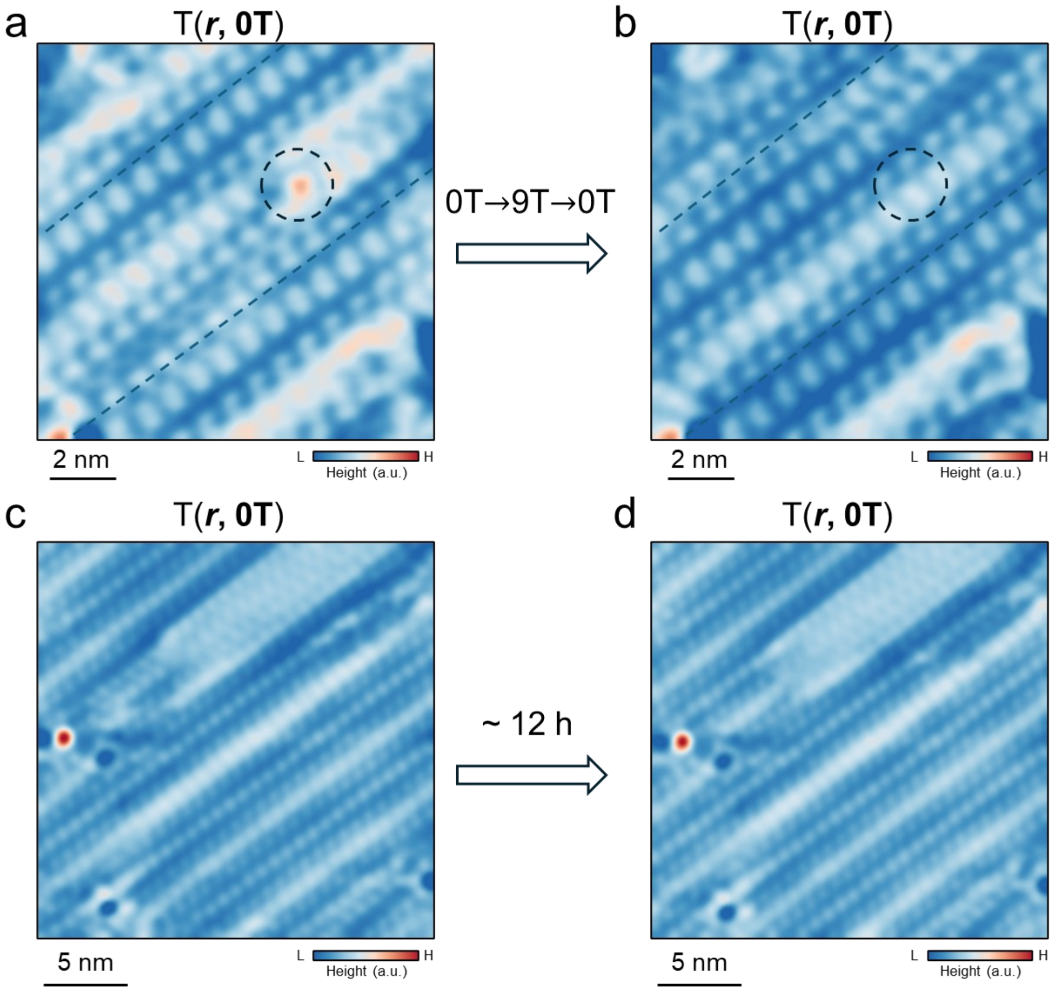

Fig. S10. Temporal stability of the CDW stripe. **a**, STM topographic image acquired at 0 T before magnetic field application. Measurement conditions : V_b=-50 mV, I=1 nA. **b**, STM topographic image at 0 T after magnetic field application. Measurement conditions : V_b=-50 mV, I=1 nA. A defect, possibly altered by the magnetic field, may induce local distortion of the stripe. **c**, **d**, Consecutive STM topographic images measured approximately 12 hours apart, demonstrating the temporal stability of the stripe configuration in the absence of magnetic field variation. Measurement conditions : (c) V_b=-67 mV, I=0.5 nA. (d), V_b=-68 mV, I=1 nA.

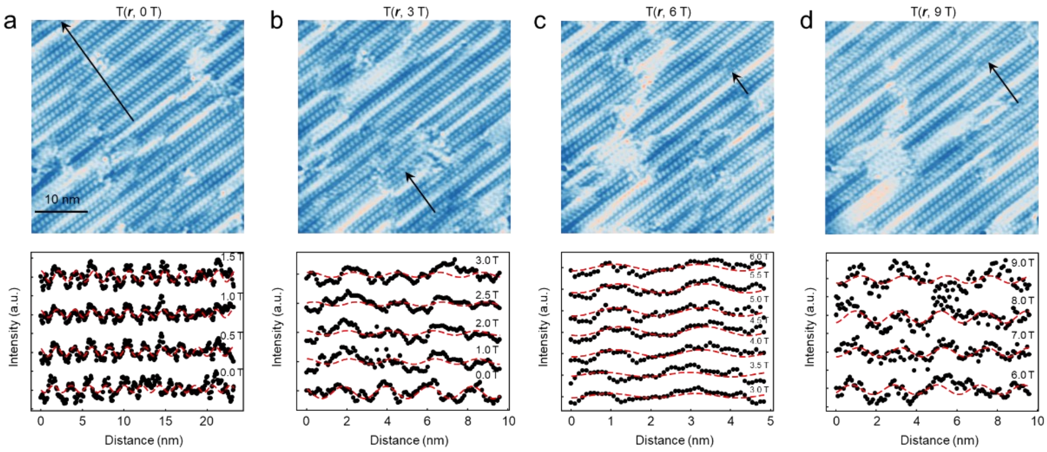

Fig. S11. Fitting of CDW phase. **a-d**, Upper panels are STM topographic images T(***r***, ***B****z*) at 0, 3, 6, 9 T, where the arrows indicate the extracted line profiles for domain #1 to #4. Lower panels show the corresponding fitting curves by using $\cos(q_{4a_0} \cdot r + \varphi)$.

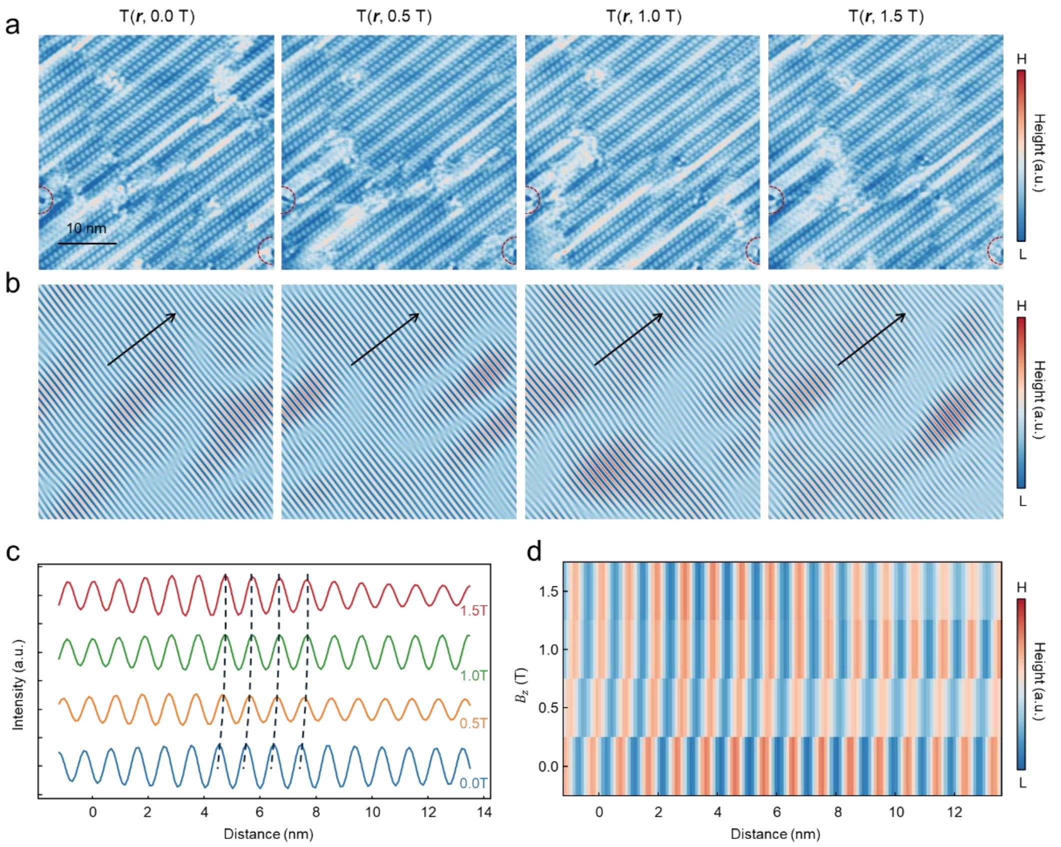

Fig. S12. Magnetic field-tunable $\sqrt{3}a_0$-CDW. **a**, STM topographies of the same field of view on Sb surface under different magnetic fields, showing the evolution of $\sqrt{3}a_0$ stripes. Measurement conditions: V_b=-50 mV, I=1 nA **b**, $\sqrt{3}a_0$-Filtered topographies of (a) by isolating the $q_{\sqrt{3}a_0}$ scattering points and performing inverse FT. **c**, Line profiles along the black arrows in (b). The dotted lines indicate the stripe shift. The datasets are vertically placed for clarity. **d**, Color map of (c).

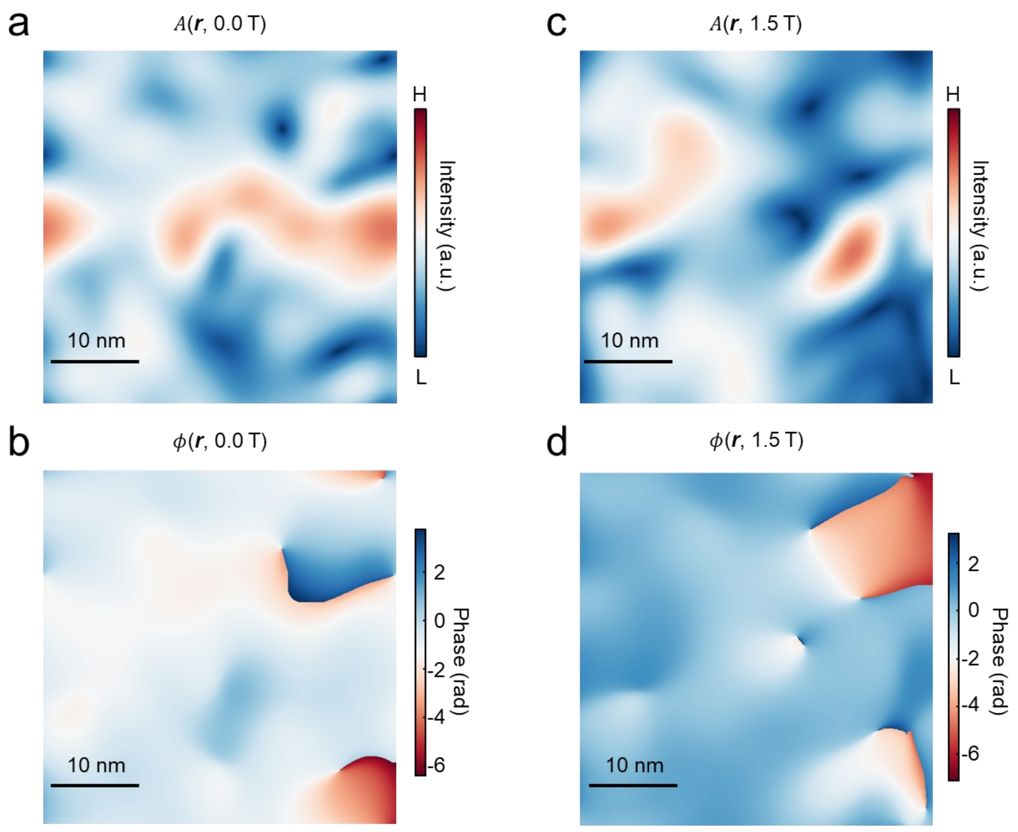

Fig. S13. 2d lock-in analysis of magnetic dependent $\sqrt{3}a_0$-CDW stripes. a-b, Spatial distribution of amplitude (a) and phase (b) of $\sqrt{3}a_0$ CDW stripes under B_z of 0.0T, extracted using 2d lock-in technique. **c-d**, Spatial distribution of amplitude (c) and phase (d) of $\sqrt{3}a_0$ CDW stripes under B_z of 1.5T, extracted using 2d lock-in technique. The average length of lock-in technique is 3.18 nm.

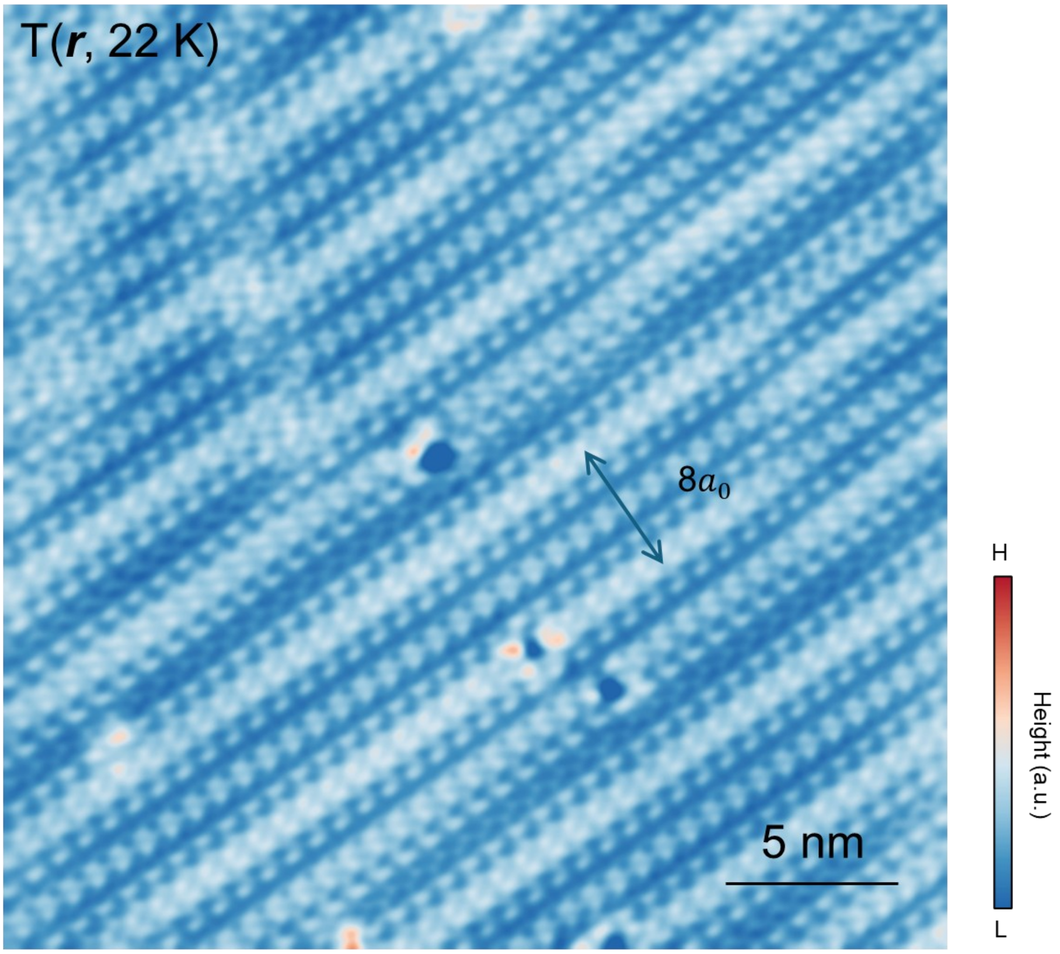

Fig. S14. Persistence of the CDW at 22 K. STM topographic image of the $8a_0 \times \sqrt{3}a_0$ CDW measured at 22 K, demonstrating its stability at 22 K. Measurement conditions: V_b=-50 mV, I=100 pA, T=22 K.

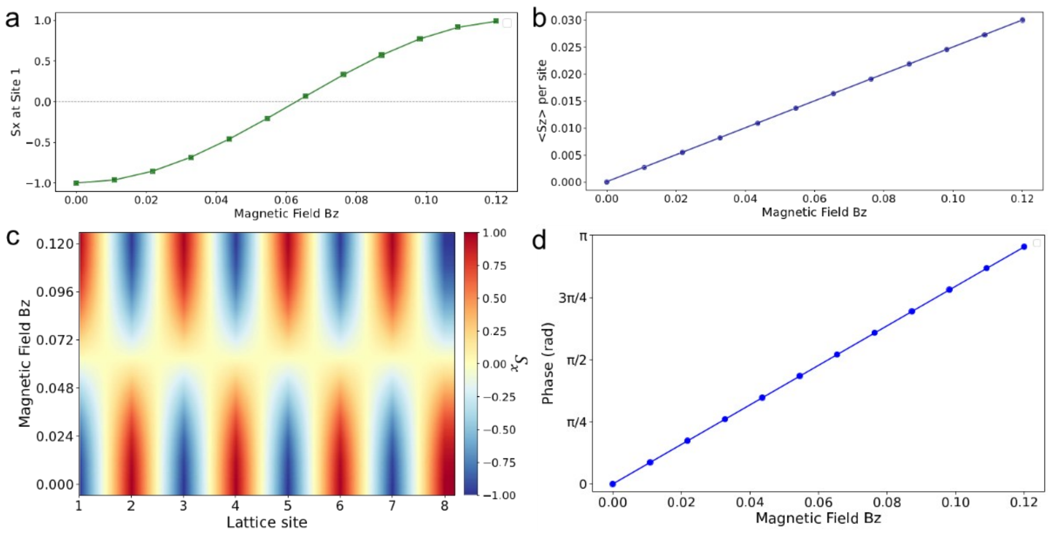

Fig. S15. LLG simulation of antiferromagnetic Heisenberg Model. a, **b**, The evolution of spin components S_x and S_z under magnetic field. The evolution of S_x is demonstrated locally at a single lattice site (site 1) as a function of both time and magnetic field, while S_z is illustrated as the spatial average over all lattice sites. **c**, The spatial distribution of spin components S_x (in-plane SDW) under magnetic field at the steady state. **d**, Phase evolution of S_x (in-plane SDW) corresponding to (**c**) under magnetic field. For simplicity, we illustrate our simulations with exchange interaction J = -1 and out-of-plane magnetic field Bz $\in [0, 0.12]$ (in units of $\frac{1}{2}J$).

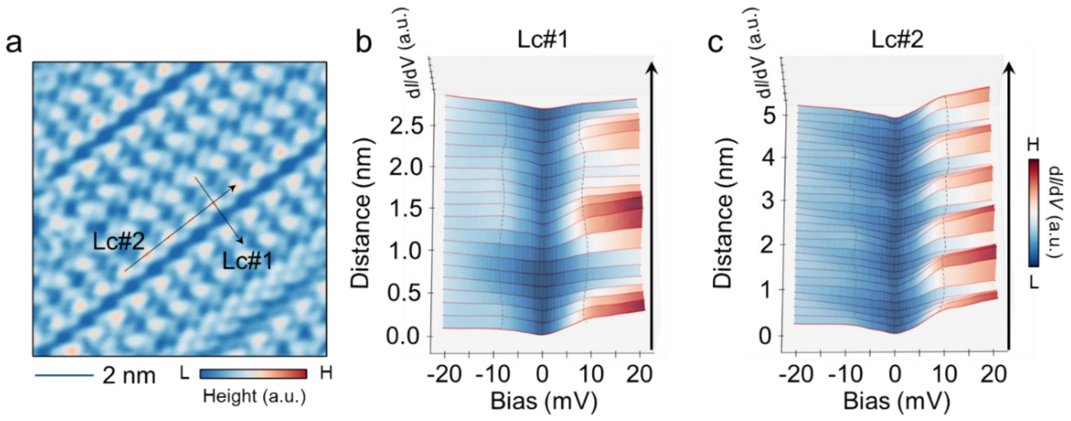

Fig. S16. Evolution of the gap feature with CDW pattern. a, STM topography, showing the CDW pattern. Measurement conditions: V_b=30 mV, I=500 pA. **b**, dI/dV line cut #1 acquired across a CDW stripe (along arrow #1 in (**a**)), showing gap variations correlated with the CDW modulation. Measurement conditions: V_b=-50 mV, I=2 nA, $V_{lock\text{-}in}$=0.5 mV. **c**, dI/dV line cut #2 acquired along the CDW stripe (along arrow #2 in (**a**)), showing gap variations correlated with the CDW modulation. Measurement conditions: V_b=-50 mV, I=2 nA, $V_{lock\text{-}in}$=0.5 mV.

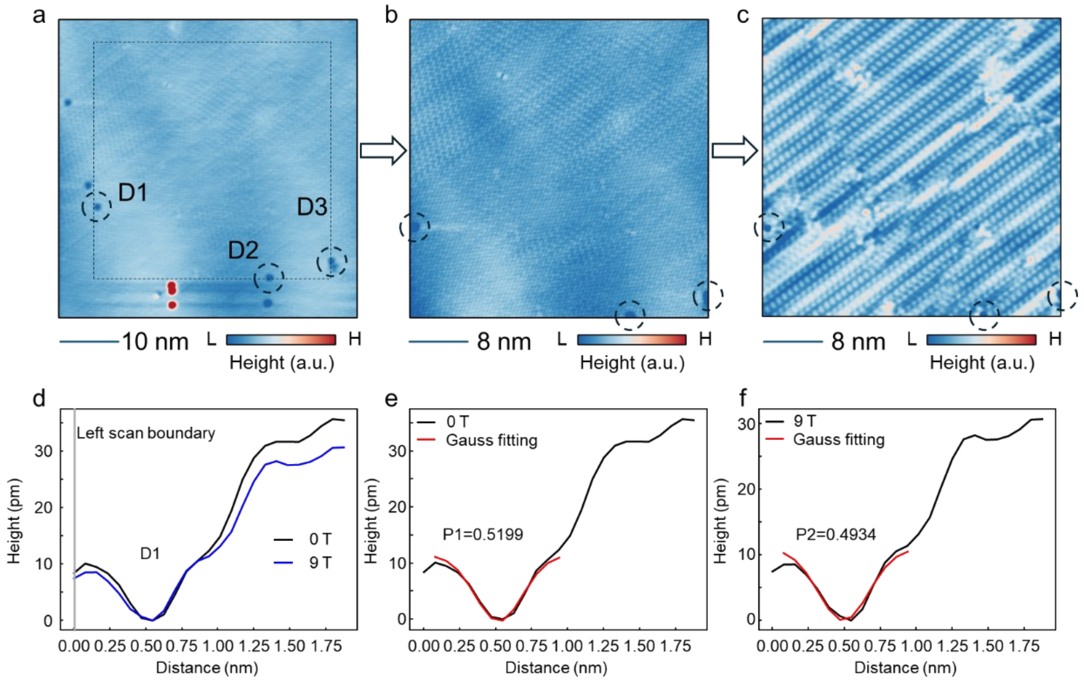

Fig. S17. Field of view alignment procedure. a, Large-scale STM topography acquired at high bias, showing several atomic lattice defects. Measurement conditions: V_b=-900 mV, I=1 nA. **b**, Smaller-scale STM topography at high bias. The field of view is aligned by matching its left edge to the left edge of defect D1, and its lower and right edges to the centers of defects D2 and D3, respectively, as indicated in (**a**). Measurement conditions: V_b=-900 mV, I=1 nA. **c**, STM topography with the same field of view as (**b**), acquired at lower bias to clearly resolve the CDW stripe pattern. Measurement conditions: V_b=-50 mV, I=1 nA. **d**, Line profiles of D1 extracted at 0 T and 9 T, respectively, showing negligible mismatch after applying our alignment procedure. The line profiles are extracted from V_b=-900 mV images. **e**-**f**, Gaussian fits to the corresponding line profiles at 0 T and 9 T, respectively, yielding an estimated alignment uncertainty of ~26 pm.

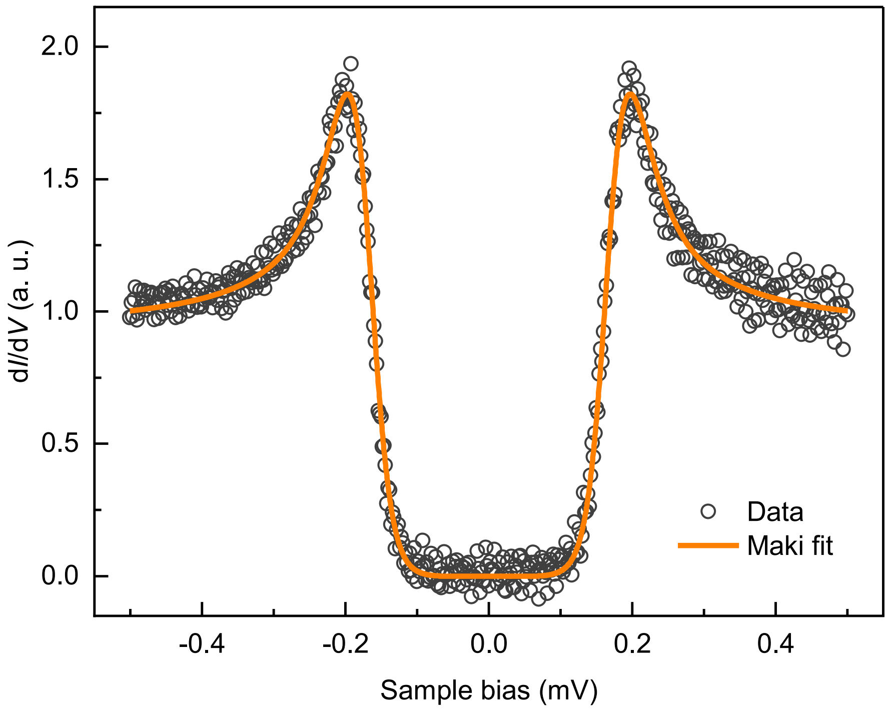

Fig. S18. Temperature calibration for dilution refrigerator STM system at the base temperature of ~50 mK. The spectrum was measured on a superconducting Al bulk polycrystal using lock-in technique with an amplitude of 3 μeV at the frequency of 726 Hz. Fitting the superconducting spectrum with BCS-Maki model gives an effective temperature of 155.7 ± 2.3 mK, superconducting gap Δ = 180.9 ± 0.5 meV, and pair-breaking parameter ζ = 0.021 ± 0.005.

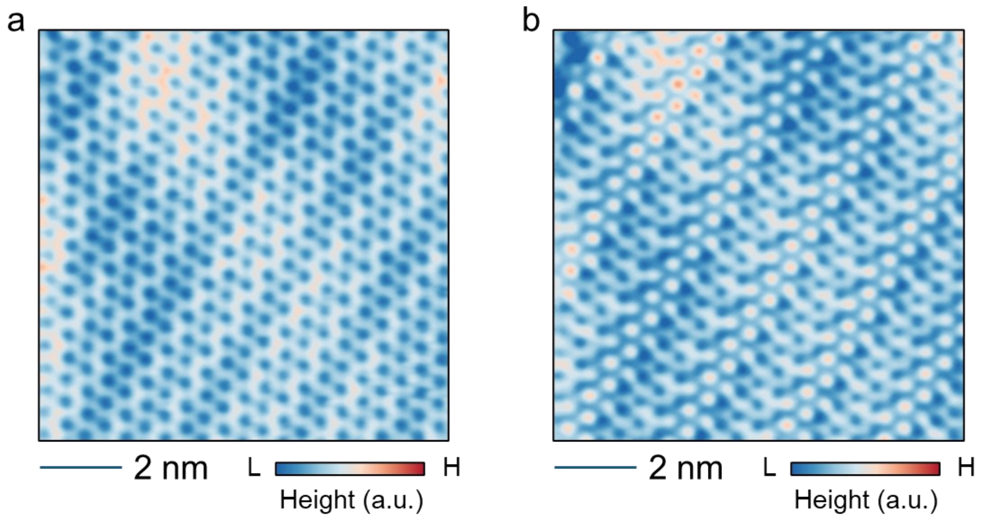

Fig. S19. STM topography of the same field of view under different bias conditions. **a**, STM topography acquired at a high sample bias of −900 mV. Measurement conditions: V_b=-900 mV, I=1 nA. **b**, STM topography acquired at a low sample bias of −50 mV. Measurement conditions: V_b=-50 mV, I=1 nA.

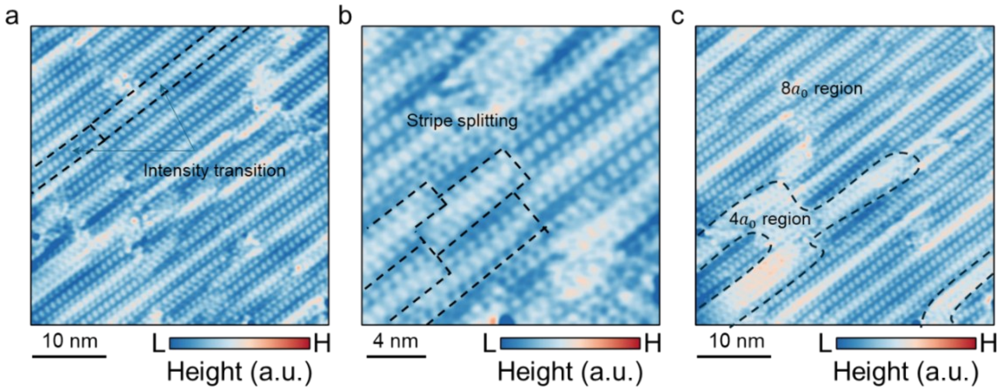

Fig. S20. Criteria for determining domain boundaries. a, Boundary formed within a single stripe. **b**, Boundary originating from stripe splitting. **c**, Boundary between domains with $4a_0$ and $8a_0$.

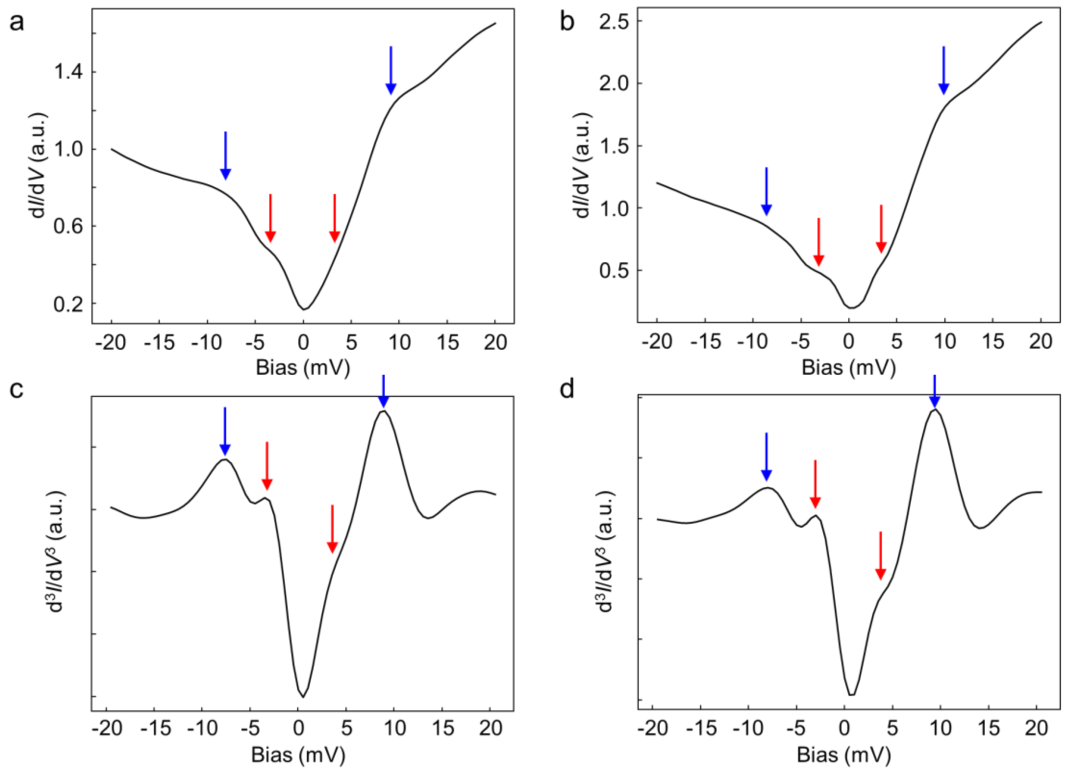

Fig. S21. Two gap features. **a**, Averaged dI/dV spectrum of the dataset in Fig. 1**g**. Measurement conditions: V_b=-50 mV, I=2 nA, $V_{lock\text{-}in}$=0.5 mV. **b**, Averaged dI/dV spectrum from an independent dataset, revealing a pair of kink features inside the primary gap. Measurement conditions: V_b=-50 mV, I=2 nA, $V_{lock\text{-}in}$=0.5 mV. **c**, Averaged d^3I/dV^3 spectrum from (**a**), showing an additional gap-like feature around Fermi level inside the 9 meV gap. **d**, Averaged d^3I/dV^3 spectrum from (**b**), revealing a pair of kink features inside the primary gap.

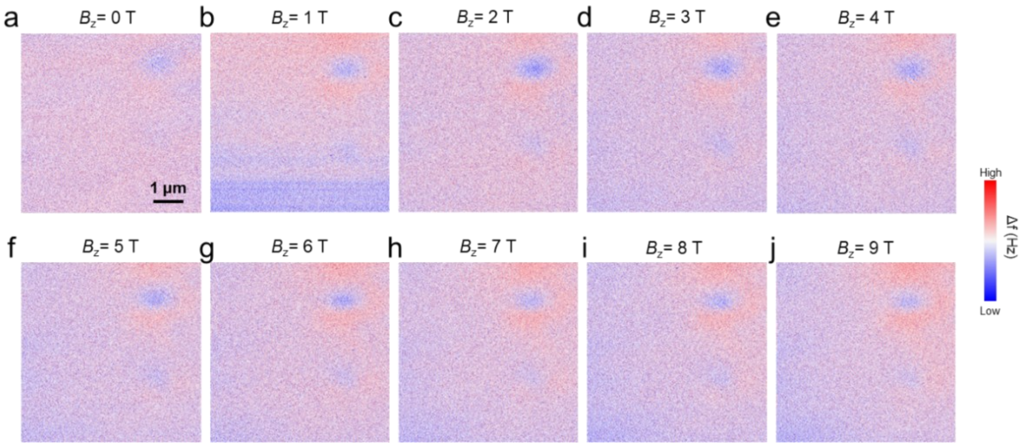

Fig. S22. Magnetic force microscopy measurements under different magnetic fields at 50 mK. **a-j**, Spatial maps of the frequency shift (Δf) acquired at B_z of 0-9 T. No discernible field-induced evolution of the Δf signal was detected across the entire investigated field range. The defect area in the upper right (small blue region) serves as a reference point for scanning the same field of view.

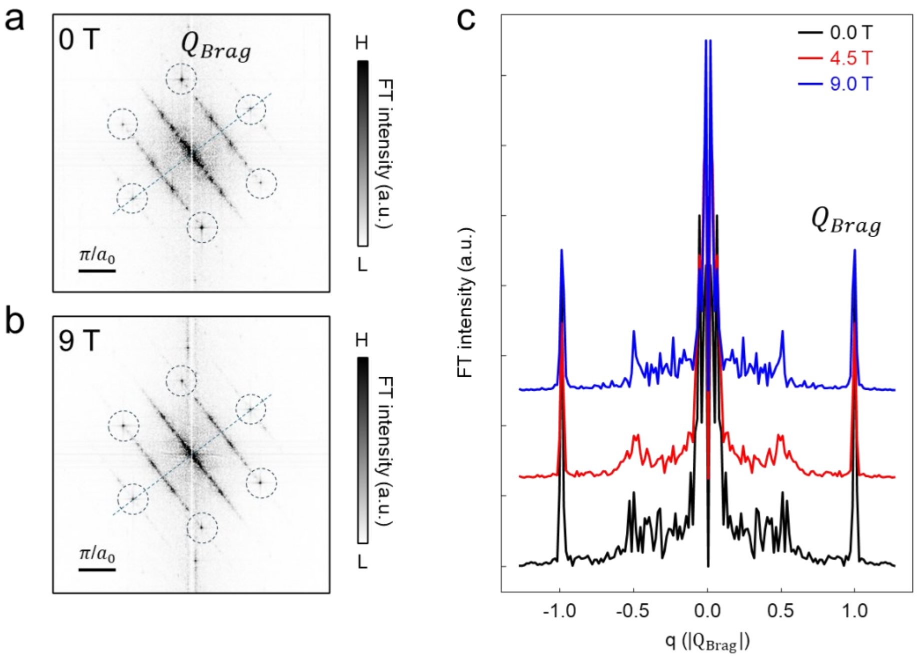

Fig. S23. Inspection of piezo-magnetic response. a, Fourier transform (FT) of the STM topography at 0 T (same field of view as Fig. 4**a**), showing Bragg peaks. Measurement conditions: V_b=-50 mV, I=1 nA. **b**, FT of the STM topography at 9 T (same field of view), showing Bragg peaks. Measurement conditions: V_b=-50 mV, I=1 nA. **c**, Comparison of the Bragg wavevector magnitude $|Q_{Brag}|$ under different magnetic field, indicating no detectable change in lattice constant within the experimental resolution. The FT line profiles were extracted along the dashed lines marked in (**a**) and (**b**).

Supplementary Table 1: Magnetic moment magnitude at each Cr site

	Magnetic Moment (μ_B)
Cr1	1.56
Cr2	1.51
Cr3	1.61
Cr4	1.38
Cr5	1.73
Cr6	1.52
Cr7	1.67
Cr8	1.36
Cr9	1.55
Cr10	1.75
Cr11	1.68
Cr12	1.70

References

1. Xu, C. *et al.* Altermagnetic ground state in distorted kagome metal $CsCr_3Sb_5$. *Nat. Commun.* **16**, 3114 (2025).
2. Li, Y. *et al.* Exotic surface stripe orders in correlated kagome metal $CsCr_3Sb_5$. Preprint at https://doi.org/10.48550/arXiv.2510.12888 (2025).
3. He, X., Helbig, N., Verstraete, M. J. & Bousquet, E. TB2J: a python package for computing magnetic interaction parameters. *Comput. Phys. Commun.* **264**, 107938 (2021).
4. Liu, Y. *et al.* Superconductivity under pressure in a chromium-based kagome metal. *Nature* **632**, 1032–1037 (2024).
5. Maki, K. Pauli paramagnetism and superconducting state. II. *Prog. Theor. Phys.* **32**, 29–36 (1964).
6. Assig, M. *et al.* A 10 mK scanning tunneling microscope operating in ultra high vacuum and high magnetic fields. *Rev. Sci. Instrum.* **84**, 33903 (2013).
7. Balashov, T., Meyer, M. & Wulfhekel, W. A compact ultrahigh vacuum scanning tunneling microscope with dilution refrigeration. *Rev. Sci. Instrum.* **89**, 113707 (2018).
8. Machida, T., Kohsaka, Y. & Hanaguri, T. A scanning tunneling microscope for spectroscopic imaging below 90 mK in magnetic fields up to 17.5 T. *Rev. Sci. Instrum.* **89**, 93707 (2018).

9. Que, Y. *et al.* Performance benchmarking of an ultra-low vibration laboratory to host a commercial millikelvin scanning tunnelling microscope. *Nanotechnology* **34**, 455704 (2023).

10. Kazakova, O. *et al.* Frontiers of magnetic force microscopy. *J. Appl. Phys.* **125**, 60901 (2019).

11. Hartmann, U. Magnetic force microscopy. *Annu. Rev. Mater. Sci.* **29**, 53–87 (1999).
