# Supplementary material for: Controlling an altermagnetic spin density wave in the kagome magnet CsCr3Sb5
Source: Nat Commun. 2026 Jun 1;17:7041. doi: 10.1038/s41467-026-73976-3 (PMC13392461; doi:10.1038/s41467-026-73976-3)
Supplement: Supplementary file 2 — Transparent Peer Review file [file 41467_2026_73976_MOESM2_ESM.pdf]

nature portfolio

Peer Review File

Controlling an altermagnetic spin density wave in the kagome magnet $CsCr_3Sb_5$

Corresponding Author: Professor Bent Weber

This file contains all reviewer reports in order by version, followed by all author rebuttals in order by version.

Version 0:

Reviewer comments:

Reviewer #1

(Remarks to the Author)
In their manuscript, the authors present a comprehensive experimental investigation of charge density wave (CDW) observation and manipulation in the Kagome magnet CsCr3Sb5, complemented by first-principles calculations. Using scanning tunneling microscopy (STM), they identify two distinct CDW configurations with periodicities of 4a and 8a. They demonstrate that both the domains of these CDW types and the phase configurations of 4a CDW stripes can be controllably modified through magnetic field application. Their first-principles simulations further reveal an altermagnetic ground state in CsCr3Sb5. While the findings regarding magnetic field-induced CDW manipulation are compelling, the current discussion of the underlying mechanisms remains underdeveloped. After the authors strengthen this aspect of the discussion and address the following points, I will be able to reach a final decision:

Major issues:

1) The authors attribute a 9 meV CDW gap to kinks observed in the dI/dV spectrum (Fig. 1g). However, close inspection of the large-scale dI/dV spectrum in Fig. 1e reveals a pronounced suppression of spectral weight spanning approximately -0.4 V to 0.4 V, centered around zero bias. This broader feature raises questions about the validity of isolating a narrow energy range (-0.01 V to 0.01 V) to assign the kinks solely to a CDW gap. The authors should provide more convince evidence and discussions about CDW gap

2) The reported field-induced CDW sliding hinges on precise spatial alignment of the imaging area across different magnetic fields. While circular markers are visible in the images (Fig. 3), their purpose is not clarified in the text. Given the subtlety of the observed CDW phase shifts, minor misalignments between successive scans could artificially produce similar patterns. To strengthen this claim, I recommend including simultaneously acquired low-bias topographs (where CDW features are prominent) alongside the high-bias images (presumably field-insensitive) as an internal control. This would conclusively demonstrate that the field-dependent changes arise from genuine CDW reconfiguration rather than instrumental drift or misregistration.

Minor issues:

1) The manuscript reports an effective temperature of 150 mK in their STM, but provides no details regarding the calibration methodology or supporting experimental data. The authors should add the experimental data and discussion to the supplementary materials

2 While the authors represent honeycomb lattice features observed at high bias with striped structures at low bias, they fail to present corresponding high and low bias images of the identical field of view. I believe such data is essential.

3 The domain boundaries marked by dotted lines in Fig. 4a appear arbitrary given the complexity of the STM image. The transition between 4a and 8a CDW domains is not clearly discernible, and the criteria used for boundary delineation are unspecified. The authors should either employ quantitative analysis to objectively identify domains, or provide higher resolution/more definitive images where the domain separation is visually unambiguous.

Reviewer #2

(Remarks to the Author)
The authors employ STM to identify two distinct linear CDWs and show that the domain walls associated with each undergo gradual amplitude and phase shifts when an external magnetic field is applied. Their DFT analysis reveals that one of these CDWs has its origin in an altermagnetic SDW, which accounts for the observed magnetic-field-induced evolution. The subject of the work is compelling and timely, and the experimental data are of excellent quality. After clarifying a few issues, the work can be published in nature communications.

1) Given that the CDW consists of two structural components, one would naturally expect two associated energy gaps. Nevertheless, the dI/dV spectra present only a single gap of 9 meV.
2) Ref. 16 reports a different ground state (4 × 2) based on DFT. Could the authors clarify how unstable the 4 × 2 configuration appears within their own calculations?
3) In Ref. 50, magnetic force microscopy was employed to map the magnetic response and compare it with the CDW pattern. A similar experimental measurement of the magnetic signal would significantly enhance the credibility of the authors' interpretation.
4) More cases have been reported in which the CDW responds to an external magnetic field. The relevant references are provided below.
Xing et al. https://doi.org/10.1038/s41586-024-07519-5
Ichicawa et al. https://doi.org/10.1038/s41699-025-00584-y
5) Xing et al. put forward electro-striction and piezo-magnetic responses as possible mechanisms, rather than the authors' stated interpretation. These appear to explain the experimental results more simply.

Version 1:

Reviewer comments:

Reviewer #1

(Remarks to the Author)
The authors have addressed my concerns,now I recomend the manuscript for publication.

Reviewer #2

(Remarks to the Author)
The authors have generally provided appropriate responses to the issues I raised. I recommend this manuscript for publication.

Open Access This Peer Review File is licensed under a Creative Commons Attribution 4.0 International License, which permits use, sharing, adaptation, distribution and reproduction in any medium or format, as long as you give appropriate credit to the original author(s) and the source, provide a link to the Creative Commons license, and indicate if changes were made.

In cases where reviewers are anonymous, credit should be given to 'Anonymous Referee' and the source.
The images or other third party material in this Peer Review File are included in the article’s Creative Commons license, unless indicated otherwise in a credit line to the material. If material is not included in the article’s Creative Commons license and your intended use is not permitted by statutory regulation or exceeds the permitted use, you will need to obtain permission directly from the copyright holder.
To view a copy of this license, visit https://creativecommons.org/licenses/by/4.0/

Responses to the reviewers' comments

We thank both reviewers for their thoughtful evaluation and positive assessment of our work. Their constructive comments have further strengthened the manuscript. In particular, we appreciate Reviewer #2's encouraging assessment that our work is "compelling and timely" and that the experimental data are of "excellent quality". Also Reviewer #1 describes our results as "compelling". We thank both reviewers for their insightful and constructive comments aimed at strengthening the discussion of the underlying CDW manipulation mechanisms and clarifying technical aspects of the experiments, which we address in our point-by-point reply below. We believe that the revisions have significantly strengthened the manuscript, and we hope that our responses and improvements will address the reviewers' concerns.

Summary of major changes to the manuscript:

- We added new Fig. S15 and spin dynamics simulation as Section A in the Supplementary Information, and discuss them in the manuscript main text.
- We added new Fig. S16 and Section B in the Supplementary Information to discuss the spectral gap features of the CDW order.
- We added new Fig. S17 and Section C in the Supplementary Information to explain the field-of-view alignment procedure.
- We added new Fig. S18 and and Section D in the Supplementary Information to show the effective electron temperature of the STM.
- We added new Fig. S19 to provide a comparison of images taken at high bias and low bias in an identical field of view.
- We added new Fig. S20 and Section E in the Supplementary Information to clarify the criteria for determining domain boundaries. We revised Fig. 4 for the domain boundaries.
- We added new Fig. S21 and incorporated the discussion of two gap features into Section B of the Supplementary Information. We also briefly discussed them in the main text.
- We added new Fig. S22 and Section F in the Supplementary Information to discuss the magnetic force microscopy measurement, which further excludes the magnetic phase transition as the origin of the magnetic-field-induced sliding CDW. The author list was also updated for the MFM measurements.
- We added discussion for more cases in which the CDW responds to an external magnetic field.
- We added new Fig. S23 and Section G in the Supplementary Information to exclude the piezo-magnetic response.
- We updated Dr. Yi Liu's funding acknowledgement information.

Response to the Reviewer #1

In their manuscript, the authors present a comprehensive experimental investigation of charge density wave (CDW) observation and manipulation in the Kagome magnet CsCr3Sb5, complemented by first-principles calculations. Using scanning tunneling microscopy (STM), they identify two distinct CDW configurations with periodicities of 4a and 8a. They demonstrate that both the domains of these CDW types and the phase configurations of 4a CDW stripes can be controllably modified through magnetic field application. Their first-principles simulations further reveal an altermagnetic ground state in CsCr3Sb5. While the findings regarding magnetic field-induced CDW manipulation are compelling, the current discussion of the underlying mechanisms remains underdeveloped. After the authors strengthen this aspect of the discussion and address the following points, I will be able to reach a final decision:

We thank the Reviewer for for regarding our findings compelling. In correlated electron systems where CDW and SDW instabilities coexist or compete, an external magnetic field can couple directly to the spin through the Zeeman effect, tuning the SDW order. In $CsCr_3Sb_5$, the CDW order is strongly coupled to the magnetic pattern as shown in our results, as well as recent works [Xu, C. *et al. Nat. Commun.* **16**, 3114 (2025); Li, Y. *et al. arXiv.*2510.12888 (2025)].

Consequently, we expect that a magnetic-field-manipulated spin order shall induce a corresponding CDW manipulation. From our additional theoretical analysis (below), we conclude that the magnetic-field-induced CDW manipulation is therefore mediated through the corresponding manipulation of the underlying SDW phase. The associated spin dynamics in a magnetic field can be qualitatively understood in analogy to a simple toy model based on a one-dimensional antiferromagnetic spin chain in a magnetic field.

We consider the antiferromagnetic Heisenberg spin-exchange Hamiltonian in a magnetic field B_z,

$$\mathcal{H} = -J\sum_{\langle ij\rangle}\mathbf{S}_i \cdot \mathbf{S}_j - B_z\sum_i S_i^z,$$

To describe the dynamics of the chain, we use the Landau-Lifshitz-Gilbert (LLG) equation,

$$\frac{d\mathbf{S}}{dt} = -\gamma\mathbf{S} \times \mathrm{H}_{\mathrm{eff}} + \alpha\mathbf{S} \times \frac{d\mathbf{S}}{dt},$$

with an effective field $\mathbf{H}_{\mathrm{eff},i} = -\frac{\partial\mathcal{H}}{\partial\mathbf{S}_i}$ and Gilbert damping parameter $\alpha = 0.01$.

To limit the computational load, we consider a chain of lattice sites L=200 with periodic boundary conditions. We further assume an in-plane antiferromagnetic ground state as the initial configuration, as justified by our DFT calculations, which show that the in-plane magnetic moment configuration is lower in energy by about 2 meV/f.u. compared to the out-of-plane (c-axis) configuration. Within the plane, the energy difference between different orientations is negligible (<1 meV/f.u.).

The simulation results are present in new Fig. S15 after the system was reached the steady state. An out-of-plane magnetic field not only induces precession of in-plane spins (Fig. S15**a**), but also introduces a weak ferromagnetic moment along the z-direction (Figs. S15**b**), driving a field-dependent transition from a collinear AFM state to a canted AFM phase.

Importantly, the spatial distribution of S_x (Fig. S15**c**) exhibits a cosine-wave modulation, whose phase exhibits a linear dependence on the magnetic field (Fig. S15**d**) similar to what is observed in the experiments. This trend qualitatively aligns very well with the linear variation of the CDW stripe phase observed experimentally (Fig. 5**d** of the main text).

Further, based on this simple toy model, our experimental results allow for a rough estimate of the

exchange interaction J. As observed in the experiment, the rate of phase shift in magnetic field is approximately $\pi/10$ T^{-1} (Fig. 5**d**). The calculations yield $\pi/(0.05J)$ (Fig. S15**d**), giving $J\sim$ -20 meV, taking the corresponding Zeeman energy (~0.1 meV at 1 T) into account. This estimate is consistent with the anisotropic nearest-neighbor exchange interaction J (ranging from -10meV to -40 meV) calculated for the altermagnetic SDW ground state of $CsCr_3Sb_5$, using the tb2J package [He, X. et al., Comput. Phys. Commun. 264, 107938 (2021)]. Such near-quantitative agreement is remarkable, given the simplicity of the 1D toy model in describing the SDW/CDW sliding mechanism.

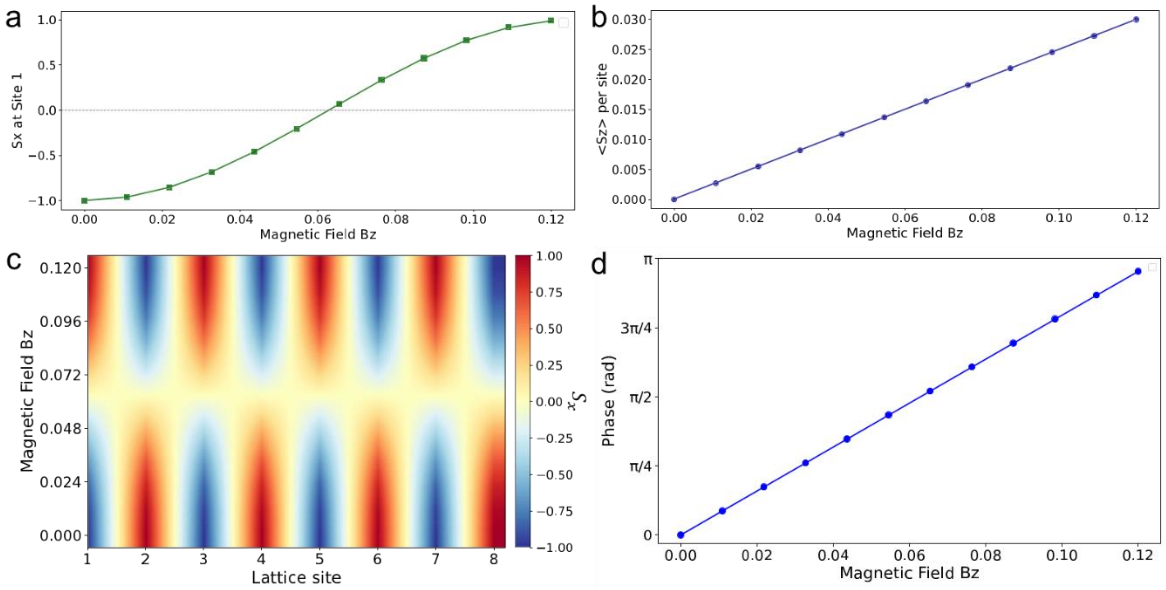

New Fig. S15. LLG simulation of antiferromagnetic Heisenberg Model. a, **b**, The evolution of spin components S_x and S_z under magnetic field. The evolution of S_x is demonstrated locally at a single lattice site (site 1) as a function of both time and magnetic field, while S_z is illustrated as the spatial average over all lattice sites. **c**, The spatial distribution of spin components S_x (in-plane SDW) under magnetic field at the steady state. **d**, Phase evolution of S_x (in-plane SDW) corresponding to (**c**) under magnetic field. For simplicity, we illustrate our simulations with exchange interaction J = -1 and out-of-plane magnetic field Bz $\in [0, 0.12]$ (in units of $\frac{1}{2}J$).

To clarify this point, we have added the following discussions in the revised manuscript:

"…Our real-space observations of field-tunable stripe displacement and amplitude variation are consistent with such a scenario. To further explore this idea, we numerically simulate the associated spin dynamics under magnetic fields using a one-dimensional antiferromagnetic chain as a minimal model for the SDW order, based on the Landau–Lifshitz–Gilbert (LLG) equation (see Supplementary Information, Section A, for details). The simulation results show that the in-plane spin component S_x (representing the in-plane SDW) under an out-of-plane field B_z exhibits a cosine-wave modulation (Fig. S15**c**), whose phase displays a linear shift with the magnetic field (Fig. S15**d**). This trend qualitatively agrees with the linear variation of the CDW stripe phase observed experimentally in Fig. 5**d**, suggesting that the magnetic-field manipulation of the CDW may originate from the magnetic field-driven modulation of the underlying SDW. It should also be noted that real material surfaces are often complicated by domain walls, defects, and strain, all of which can influence the SDW texture and its field response…"

We have also added the calculation details in the supplementary information as Section A and new

Fig. S15.

We also thank the Reviewer for his/her valuable comments and suggestions, which we will address point by point below.

Comment 1: *The authors attribute a 9 meV CDW gap to kinks observed in the dI/dV spectrum (Fig. 1g). However, close inspection of the large-scale dI/dV spectrum in Fig. 1e reveals a pronounced suppression of spectral weight spanning approximately -0.4 V to 0.4 V, centered around zero bias. This broader feature raises questions about the validity of isolating a narrow energy range (-0.01 V to 0.01 V) to assign the kinks solely to a CDW gap. The authors should provide more convince evidence and discussions about CDW gap*

We thank the Reviewer for raising this point regarding the identification of the CDW gap. The large-scale *dI/dV* spectrum shown in Fig. 1**e** is intended to present the overall electronic density of states over a wide energy range. The suppression observed in the spectrum between ±0.4 V simply arises from an overall reduction in the density of states, also reflected in our DFT calculations. In the measurement, due to the wide energy range, a relatively large lock-in amplitude ($V_{lock\text{-}in}$=10 mV) had to be used, which may have averaged over fine spectral features near the Fermi level.

Fig. 1**g** shows *high-resolution* spectroscopy within a narrow bias window, and with lock-in amplitude $V_{lock\text{-}in}$ =0.5 mV, clearly showing the pronounced spectral kinks symmetrically at ±9 meV, which we attribute to the opening of a CDW gap. The magnitude of this gap is consistent with transport measurements of $CsCr_3Sb_5$ where a CDW transition temperature of $T_{\mathrm{DW}} \approx 55$ K was recently found [Ref. 29, Liu *et al.*, *Nature* **632**, 1032 – 1037(2024)]. Using the mean-field estimate $\Delta \approx 1.76 k_{\mathrm{B}} T_{\mathrm{DW}}$, one obtains $\Delta \approx 8.4$ meV, in excellent agreement with the gap magnitude observed in our measurements.

To further substantiate the identification of the CDW gap, we have performed a dI/dV linecut analysis illustrating the spatial evolution of the gap across the CDW modulation (New Fig. S16). As shown by series of spectra taken along both perpendicular to the stripes (New Fig. S16**b**) and along the stripe direction (New Fig. S16**c**), the gap magnitude exhibits clear spatial modulations that track the CDW pattern. This direct correlation between the local gap strength and the CDW modulation provides further compelling evidence that the observed spectral kinks indeed originate from CDW order.

Taken together, the combination of high-energy-resolution tunneling spectroscopy, the spatial modulation of the gap correlated with the CDW pattern, and consistency with transport-derived energy scales provides strong support for attributing the observed ±9 meV spectral kinks to the presence of the CDW gap. Accordingly, we discuss the identification of the CDW gap in Supplementary Information as Section B.

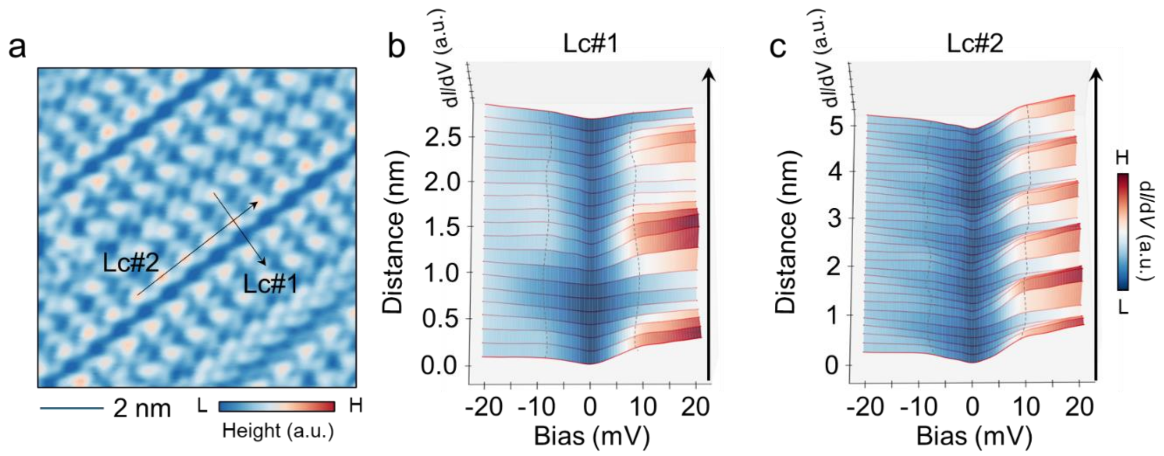

New Fig. S16. Evolution of the gap feature with CDW pattern. a, STM topography, showing the CDW pattern. Measurement conditions: V_b=30 mV, I=500 pA. **b**, dI/dV line cut #1 acquired across a CDW stripe (along arrow #1 in (**a**)), showing gap variations correlated with the CDW modulation. Measurement conditions: V_b=-50 mV, I=2 nA, $V_{lock\text{-}in}$=0.5 mV. **c**, dI/dV line cut #2 acquired along the CDW stripe (along arrow #2 in (**a**)), showing gap variations correlated with the CDW modulation. Measurement conditions: V_b=-50 mV, I=2 nA, $V_{lock\text{-}in}$=0.5 mV.

Comment 2: *The reported field-induced CDW sliding hinges on precise spatial alignment of the imaging area across different magnetic fields. While circular markers are visible in the images (Fig. 3), their purpose is not clarified in the text. Given the subtlety of the observed CDW phase shifts, minor misalignments between successive scans could artificially produce similar patterns. To strengthen this claim, I recommend including simultaneously acquired low-bias topographs (where CDW features are prominent) alongside the high-bias images (presumably field-insensitive) as an internal control. This would conclusively demonstrate that the field-dependent changes arise from genuine CDW reconfiguration rather than instrumental drift or misregistration.*

We thank the Reviewer for raising this point regarding spatial alignment and for the constructive suggestion to use high-bias images as an internal control. The circular markers shown in Fig. 3 denote the same three atomic-scale defects, which serve as fixed reference points to precisely align the imaging area across different magnetic fields. These lattice defects are field-insensitive and remain unchanged throughout the measurements, allowing us to reliably track the same region of the surface with picometer precision. We now clarify the role of the circular markers in the main text and figure captions:

"**Fig. 4. Magnetic-field tunability of CDW domains**. **a**, STM topographic images T($\boldsymbol{r}$, $\boldsymbol{B}_z$) of a mixed $4a_0 \times \sqrt{3}a_0$ and $8a_0 \times \sqrt{3}a_0$ CDW region under out-of-plane fields B_z at 0, 3, 6, and 9 T. The semi-circles mark atomic lattice defects used as alignment references and dotted lines mark CDW domains. Measurement conditions: V_b=-50 mV, I=1 nA."

To further ensure that the observed CDW phase shifts are intrinsic and not caused by instrumental drift or image misregistration, we had implemented the following procedures at each magnetic field:

1. **Continuous tip engagement:** The STM tip is not withdrawn during magnetic field ramping,

which significantly suppresses piezo-related drift associated with tip retraction and re-approach.

2. **Post-field stabilization:** After reaching the target magnetic field, we allow sufficient time for the STM to thermally and mechanically stabilize before imaging.

3. **High-bias reference imaging:** We first acquire a relatively large field-of-view topograph at high bias (New Fig. S17**a**), where the CDW contrast is weak allowing atomic defects to be clearly identified. These high-bias images, together with the defect markers, serve as an internal reference to confirm spatial alignment across fields.

4. **Precise field-of-view registration:** Guided by the defect positions in the high-bias images, we then reduce the scan area and carefully align the field of view (New Fig. S17**b**) such that its boundaries are fixed relative to the three reference defects (D1–D3), ensuring consistent positioning for all low-bias scans (New Fig. S17**c**).

This procedure indeed includes a comparison of high-bias and low-bias data (Step 3, New Figs. S17**b** and S17**c**), as suggested by the Reviewer, confirming precise alignment for each applied magnetic field. This ensures that any residual drift is negligible compared to the observed CDW phase shifts. We can estimate the alignment uncertainty by comparing the position of defect D1 at different magnetic fields, as demonstrated in new Figs. S17**d-f**. As shown, the positions of D1 at 0 T and 9 T exhibit negligible mismatch away from the left scan boundary (Fig. S17**d**). Gaussian fits yield an estimated alignment uncertainty of approximately 26 pm (Figs. S17**e-f**). Given that $4a_0$~2200 pm, the relative alignment uncertainty is about 1%.

Aside, we note that, as shown in the Fig. S8, the CDW stripes undergo pronounced field-induced reconfigurations while the reference defects remain spatially fixed. This directly demonstrates that the observed phase evolution originates from genuine CDW sliding rather than instrumental drift or scan misregistration. We have now added a note in supplementary information as Section C.

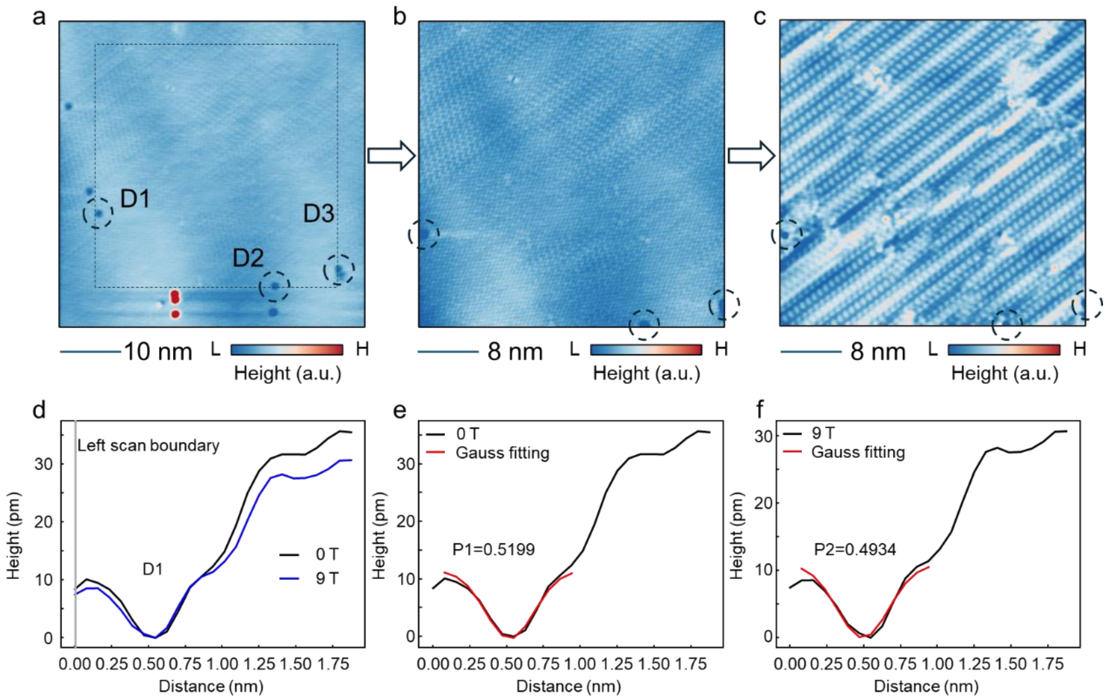

New Fig. S17. Field of view alignment procedure. a, Large-scale STM topography acquired at high bias, showing several atomic lattice defects. Measurement conditions: V_b=-900 mV, I=1 nA. **b**, Smaller-scale STM topography at high bias. The field of view is aligned by matching its left edge to the left edge of defect D1, and its lower and right edges to the centers of defects D2 and D3, respectively, as

indicated in (**a**). Measurement conditions: V_b=-900 mV, I=1 nA. **c**, STM topography with the same field of view as (**b**), acquired at lower bias to clearly resolve the CDW stripe pattern. Measurement conditions: V_b=-50 mV, I=1 nA. **d**, Line profiles of D1 extracted at 0 T and 9 T, respectively, showing negligible mismatch after applying our alignment procedure. The line profiles are extracted from V_b=-900 mV images. **e-f**, Gaussian fits to the corresponding line profiles at 0 T and 9 T, respectively, yielding an estimated alignment uncertainty of ~26 pm.

Comment 3: *The manuscript reports an effective temperature of 150 mK in their STM, but provides no details regarding the calibration methodology or supporting experimental data. The authors should add the experimental data and discussion to the supplementary materials*

We thank the reviewer for the suggestion. The effective electron temperature of our STM T_{eff} ~150 mK has previously been determined by spectroscopic measurement of a superconducting aluminum (Al) polycrystal and was reported in [Que et al. Nanotechnology 34, 455704 (2023)]. More recent measurement data is shown in a new Fig.S18. Fitting the superconducting LDOS with the BCS-Maki model we find $T_{\text{eff}} = 155.7 \pm 2.3$ mK, confirming our previous measurements. The corresponding energy resolution ($3.5k_B T_{\text{eff}}$) of ~46 μeV is significantly greater than the characteristic energy scales of the charge density wave phenomena (~9 meV) studied in this work.

We now include a reference to our previous work [Que et al. Nanotechnology 34, 455704 (2023)] as well as add the additional data and fits in Supplementary Section D.

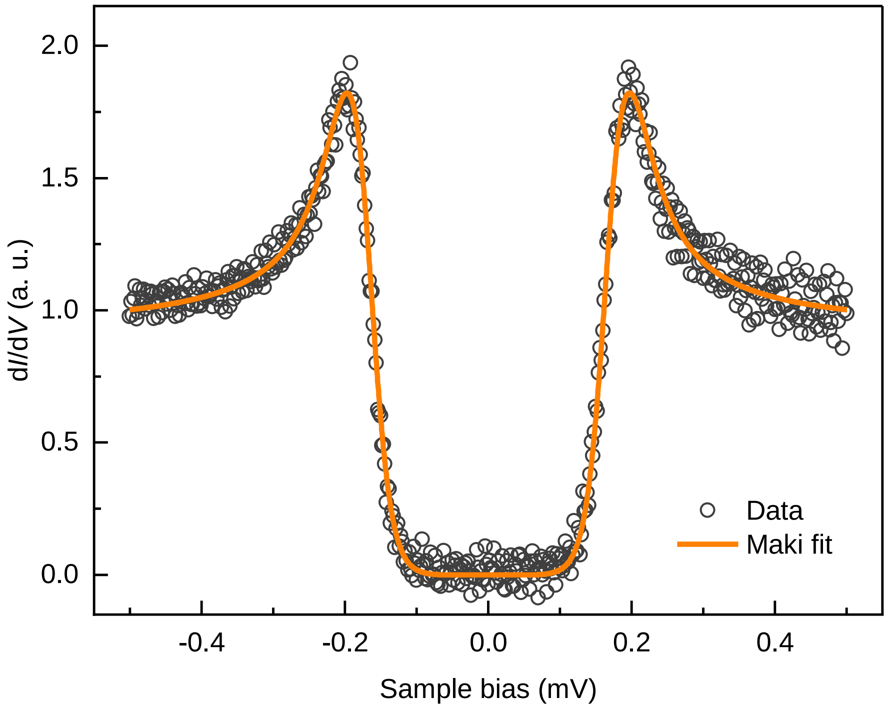

New Fig. S18. Temperature calibration for dilution refrigerator STM system at the base temperature of ~50 mK. The spectrum was measured on a superconducting Al bulk polycrystal using lock-in technique with an amplitude of 3 μeV at the frequency of 726 Hz. Fitting the superconducting spectrum with BCS-Maki model gives an effective temperature of 155.7 ± 2.3 mK, superconducting gap Δ = 180.9 ± 0.5 meV, and pair-breaking parameter ζ = 0.021 ± 0.005.

Section D. Calibration of the effective electron temperature

The effective electron temperature (T_{eff}) of the scanning tunneling microscope (STM) in our dilution refrigerator was calibrated in situ using spectroscopic measurements on a superconducting aluminum (Al) bulk polycrystal. Prior to the spectroscopic measurement, the Al polycrystal surface was cleaned through a few cycles of argon ions sputtering followed by annealing at ~350 ˚C. Following this preparation, the sample was transferred directly into the STM head at UHV conditions without exposure to ambient atmosphere. The measured density of states is influenced by thermal broadening, characterized by T_{eff}. To extract this parameter, the data were fitted using the Maki-BCS model for superconductivity in the presence of pair-breaking effects [Maki K. Prog. Theor. Phys. 32 29–36(1964); Assig M et al. Rev. Sci. Instrum. 84 033903 (2013); Balashov T et al. Rev. Sci. Instrum. 89 113707 (2018); Machida T et al. Rev. Sci. Instrum. 89 093707 (2018); Que YD et al. Nanotechnology 34 455704 (2023)]. Within this framework, the normalized superconducting density of states $N_s(E)$ is given by:

$$\rho(E) = Re\left(\frac{u}{\sqrt{u^2 - 1}}\right)$$

$$u = \frac{E}{\Delta} + \zeta\frac{u}{\sqrt{1 - u^2}}$$

Where Δ is the superconducting energy gap and ζ is the pair-breaking parameter. To account for thermal broadening, this intrinsic density of states is convolved with the derivative of the Fermi-Dirac distribution function. The fitting procedure, with Δ, ζ, and T_{eff} as free parameters, yielded an effective temperature of $T_{\mathrm{eff}} = (155.7\ \pm\ 2.3)$ mK. A representative spectrum and the corresponding best-fit curve are shown in Supplementary Figure 18. This T_{eff} corresponds to an energy resolution ($\delta E \approx 3.5k_B T_{\mathrm{eff}}$) of ~46 μeV, which is significantly smaller than the characteristic energy scales (~9 meV) of the charge density wave phenomena investigated in this work, confirming the adequacy of our instrumental resolution.

Comment 4: *While the authors represent honeycomb lattice features observed at high bias with striped structures at low bias, they fail to present corresponding high and low bias images of the identical field of view. I believe such data is essential.*

We apologize for this omission. We now provide a direct comparison of images taken at high bias (Fig. S19**a**) and low bias (Fig. S19**b**) in the Supplementary Information. While the high-bias data predominantly shows the honeycomb lattice contrast, the low bias data clearly shows the CDW modulation in an identical field of view, as shown in the new Fig. S19 below.

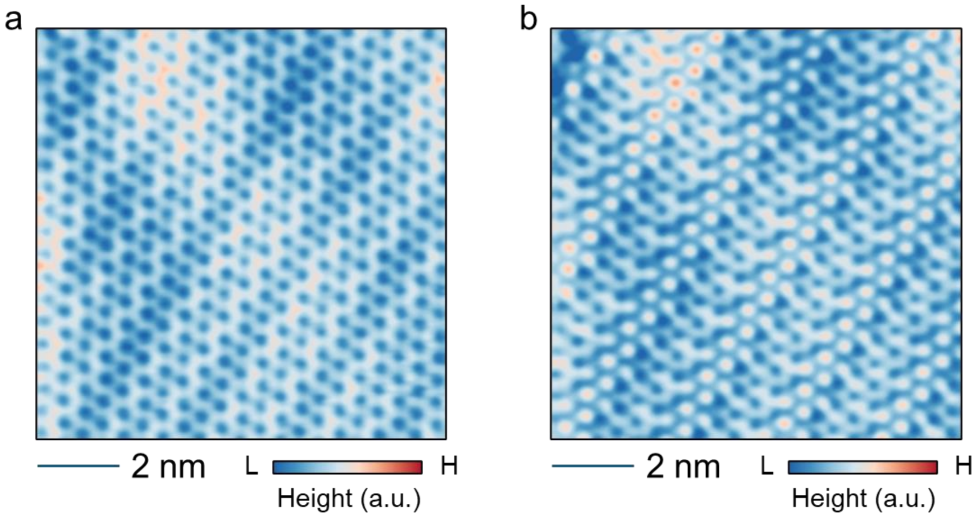

New Fig. S19. STM topography of the same field of view under different bias conditions. **a**, STM topography acquired at a high sample bias of −900 mV. Measurement conditions: V_b=-900 mV, I=1 nA. **b**, STM topography acquired at a low sample bias of −50 mV. Measurement conditions: V_b=-50 mV, I=1 nA.

Comment 5: *The domain boundaries marked by dotted lines in Fig. 4a appear arbitrary given the complexity of the STM image. The transition between 4a and 8a CDW domains is not clearly discernible, and the criteria used for boundary delineation are unspecified. The authors should either employ quantitative analysis to objectively identify domains, or provide higher resolution/more definitive images where the domain separation is visually unambiguous.*

We thank the Reviewer for the query on the identification of CDW domain boundaries. We agree that, given the complexity of the STM topography, the criteria for delineating the domain boundaries should be clearly defined.

In the previous Fig. 4**a** of the manuscript, the dotted lines are not drawn arbitrarily. Instead, they are guided by the results of the two-dimensional lock-in analysis (Figs. 4**c** and 4**d**) performed at q_{4a_0}, which provide spatial maps of the amplitude and phase of $4a_0$ modulation, respectively. These maps reveal distinct regions with different $4a_0$ modulation characteristics, from which the approximate domain boundaries were initially inferred.

Some residual uncertainty in identifying the precise location of the domain boundaries primarily arises from the intrinsic limitation of the lock-in analysis, which operates on a single q-vector (q_{4a_0}) and requires spatial averaging over 3.18 nm (Gaussian broadening). This procedure therefore smooths atomically sharp variations and obscures abrupt domain walls. A similar limitation applies to the filtered images obtained by inverse Fourier transform of the q_{4a_0} and q_{8a_0} components (Fig. 4**b**). Because the $4a_0$ and $8a_0$ modulations are harmonically related, their coexistence and gradual crossover make sharp boundaries difficult to isolate in the filtered images alone.

To address this concern, we now further identify the domain boundaries directly from the atomic-resolution STM topography images in Fig. 4**a**, as suggested by the Reviewer. Specifically, the boundaries are determined based on the following criteria (New Fig. S20):

1. **Abrupt changes in stripe intensity within individual CDW stripes**, signaling a transition

between internal structures of CDW pattern (New Fig. S20**a**).

2. **Stripe splitting or merging events**, which occur also between internal structures of CDW pattern (New Fig. S20**b**).

3. **Direct transitions between $4a_0$ and $8a_0$ stripe periodicities**, clearly visible in real space (New Fig. S20**c**).

These criteria now enable identification of atomically sharp boundaries and are overall consistent with the variations of CDW amplitude and phase obtained from the single-q lock-in analysis.

We have included Section E to clarify our procedures for determining domain boundaries.

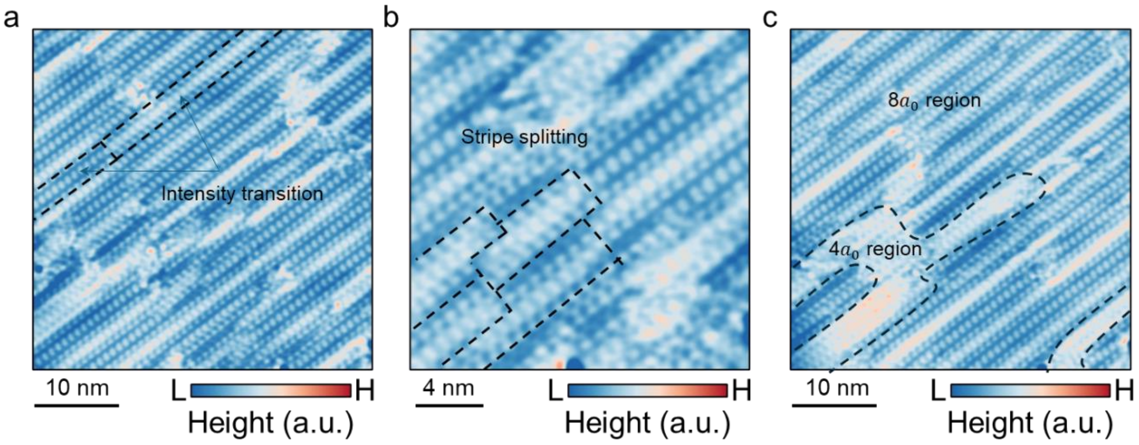

New Fig. S20. Criteria for determining domain boundaries. a, Boundary formed within a single stripe. **b**, Boundary originating from stripe splitting. **c**, Boundary between domains with $4a_0$ and $8a_0$.

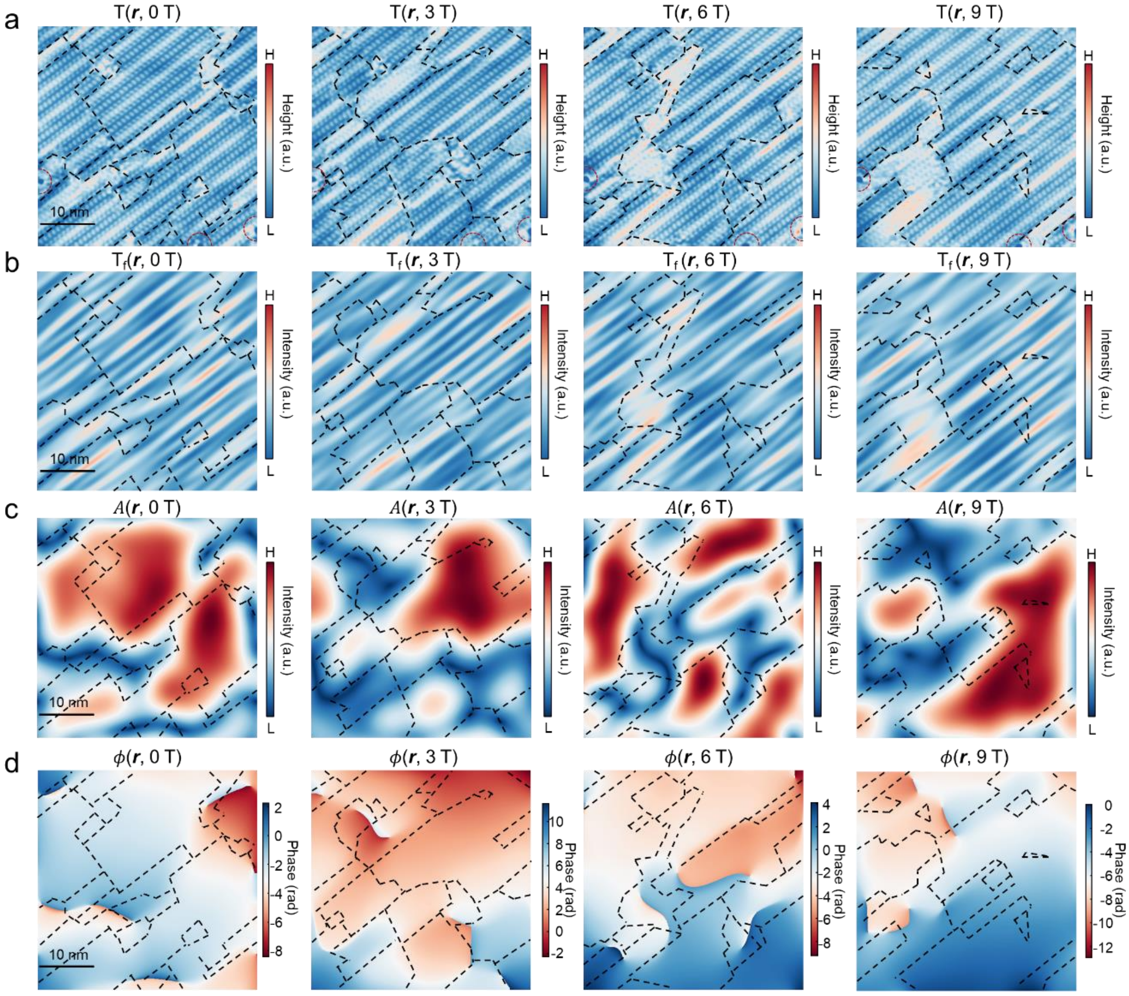

Revised Fig. 4. Magnetic-field tunability of CDW domains. a, STM topographic images T($\boldsymbol{r}$, $\boldsymbol{B}_z$) of a mixed $4a_0 \times \sqrt{3}a_0$ and $8a_0 \times \sqrt{3}a_0$ CDW region under out-of-plane fields B_z at 0, 3, 6, and 9 T. The semi-circles mark atomic lattice defects used as alignment references and dotted lines mark CDW domains. Measurement conditions: V_b=-50 mV, I=1 nA. **b**, Filtered topographic image T_f ($\boldsymbol{r}$, $\boldsymbol{B}_z$) obtained by isolating q_{4a_0} and q_{8a_0} and performing inversed FT, from the same regions as in (a). **c**, Spatial maps of $4a_0$ stripe amplitude $A(\boldsymbol{r}, \boldsymbol{B}_z)$, extracted using 2d lock-in technique. **d**, Spatial maps of the unwrapped phase $\phi(\boldsymbol{r}, \boldsymbol{B}_z)$ of $4a_0$ stripes, extracted using 2d lock-in technique. The average length of lock-in technique is 3.18 nm.

In summary, we believe we have addressed all comments and questions raised by the Reviewer, adding new data and discussions. Once again, we sincerely thank the Reviewer for the insightful, and constructive criticism, which have helped us to further strengthen the manuscript.

Response to the Reviewer #2

The authors employ STM to identify two distinct linear CDWs and show that the domain walls associated with each undergo gradual amplitude and phase shifts when an external magnetic field is applied. Their DFT analysis reveals that one of these CDWs has its origin in an altermagnetic SDW, which accounts for the observed magnetic-field-induced evolution. The subject of the work is compelling and timely, and the experimental data are of excellent quality. After clarifying a few issues, the work can be published in nature communications.

We sincerely thank the Reviewer for the concise summary and very positive assessment of our work. We also thank the Reviewer for the valuable comments and suggestions, which we will address point by point below.

Comment 1: *Given that the CDW consists of two structural components, one would naturally expect two associated energy gaps. Nevertheless, the dI/dV spectra present only a single gap of 9 meV.*

We thank the Reviewer for this insightful comment. Indeed, given the presence of two structural components in the CDW, one may expect multiple associated energy scales in the tunneling spectra.

In Fig. 1**g**, the most prominent spectral feature is a pair of pronounced kinks at approximately ±9 meV, which we attribute to the primary CDW gap. Motivated by the Reviewer's comment, we have performed additional analysis and found a second, weaker kink structure located within the larger gap.

Our analysis is shown in New Fig. S21 for two separate data sets, the original Fig. 1**g** as well as a new data set, not previously presented. In both, we carried out a second-derivative analysis. This analysis reveals two distinct energy scales: a dominant pair of peaks corresponding to the original ±9 meV gap, and a second pair of features at lower energy, around ±3 meV. The latter may naturally be associated with the second CDW structural component.

Taken together, the second-derivative analysis and the consistency across independent measurements provide strong evidence that the two CDW structural components are associated with two distinct energy scales. We sincerely thank the Reviewer for prompting this more detailed analysis, which has further strengthened the interpretation of our spectroscopic results.

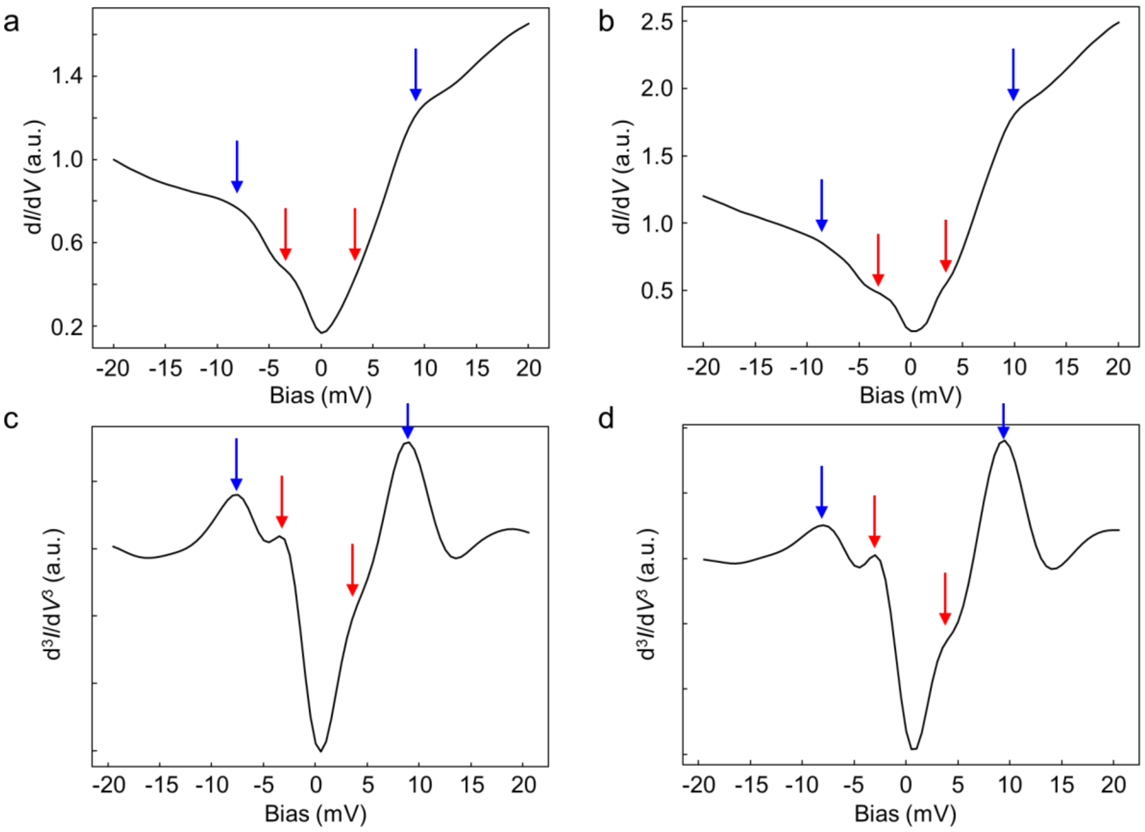

New Fig. S21. Two gap features. a, Averaged dI/dV spectrum of the dataset in Fig. 1g. Measurement conditions: V_b=-50 mV, I=2 nA, $V_{lock-in}$=0.5 mV. **b**, Averaged dI/dV spectrum from an independent dataset, revealing a pair of kink features inside the primary gap. Measurement conditions: V_b=-50 mV, I=2 nA, $V_{lock-in}$=0.5 mV. **c**, Averaged d^3I/dV^3 spectrum from (**a**), showing an additional gap-like feature around Fermi level inside the 9 meV gap. **d**, Averaged d^3I/dV^3 spectrum from (**b**), revealing a pair of kink features inside the primary gap.

We have included the discussion of this analysis into Supplementary Section B and added the following discussions in the revised manuscript:

"…we observe clear inversion of contrast upon bias reversal, consistent with the particle-hole symmetry breaking. In addition, we observe in both the dI/dV spectra and their second derivative (d^3I/dV^3) another distinct energy scale (Fig. S21): a pair of features around ±3 meV, which may be associated with the second CDW structural component."

Comment 2: *Ref. 16 reports a different ground state (4 × 2) based on DFT. Could the authors clarify how unstable the 4 × 2 configuration appears within their own calculations?*

We thank the Reviewer for raising this important question. Ref. 16 indeed reports a 4 × 2 magnetic ground state based on density functional theory calculations for the $CsCr_3Sb_5$ *bulk*. As STM is a surface sensitive technique, the present calculations reflect the electronic properties of the *surface*. To this end, the DFT calculations consider a surface model composed of a monolayer Cr_3Sb_5 with the Cs atoms

removed. This is consistent with the experiment, probing the Sb-terminated surface.

Within this framework, we had evaluated the relative stability of multiple magnetic configurations, including the 4 × 2 SDW state reported in Ref. 16. However, as shown in Fig. S6**a**, the 4 × 2 SDW configurations (indicated by square symbols) are significantly higher in energy (more than ~50 meV per unit cell), compared to the observed 4 × √3 altermagnetic SDW ground state. This is consistent with our STM-based observation of a 4 × √3 ground state charge order.

Comment 3: *In Ref. 50, magnetic force microscopy was employed to map the magnetic response and compare it with the CDW pattern. A similar experimental measurement of the magnetic signal would significantly enhance the credibility of the authors' interpretation.*

We thank the Reviewer for this suggestion and for pointing out Ref. 50, where magnetic force microscopy (MFM) was successfully used to correlate magnetic response with CDW phase transition. However, there are important differences between the magnetic phenomena probed in Ref. 50 and the altermagnetic SDW studied in our work, which limit the applicability of MFM in our case.

Specifically, MFM is primarily sensitive to stray magnetic fields arising from a net magnetization and is therefore well suited to detecting ferromagnetic or ferrimagnetic domain structures [Kazakova, O. *et al. J. Appl. Phys.* **125**, 60901 (2019); Hartmann, U. *Annu. Rev. Mater. Sci.* **29**, 53–87 (1999)]. In Ref. 50 [Han, X. *et al. arXiv*.2503.05545 (2025)], the CDW is coupled to a two-step magnetic phase transition. Near the critical field corresponding to the first transition, magnetic domains of the ground-state phase (antiferromagnetic state) and those of the 1/3 plateau phase coexist in $GdTi_3Bi_4$. The antiferromagnetic phase has zero net magnetization, whereas the 1/3 plateau phase exhibits a weak net magnetization. As a result, the two types of domains produce MFM signals with different intensities and can therefore be distinguished experimentally. Similarly, near the higher critical field associated with the second transition, the relatively weak net magnetization of the 1/3 plateau phase and the much stronger net magnetization of the forced ferromagnetic phase show a pronounced contrast. Consequently, clear magnetic domain signals can also be observed in MFM measurements.

This stands in contrast to $CsCr_3Sb_5$, whose SDW state is of an altermagnetic nature without any net magnetization and the magnetic-field-induced CDW sliding does not involve any magnetic phase transition into a ferromagnetic order. Consequently, MFM would be expected to be intrinsically insensitive to this form of magnetic order. Furthermore, the typical spatial resolution of MFM is on the scale of hundreds of nanometers, which is larger than the observed CDW domain size (tens of nanometers), further hindering effective measurement.

Nevertheless, to address the Reviewer's concern, and to further rule-out the presence of ferromagnetic domains or phase transition, we have performed low-temperature MFM down to 50 mK under varying magnetic fields. The MFM experiments were captured by a commercial magnetic force microscope (attoAFM, attocube) using a commercial magnetic tip (MFMR, Nanoword). The MFM is coupled to a closed-cycle He cryostat and dilution refrigerator system (both Bluefors), combined with a vector magnet (9-3-1, Bluefors). The scanning probe system was operated at the resonance frequency of the magnetic tip, approximately 70 kHz. The MFM signal, i.e., the resonance frequency shift (Δf), is proportional to the out-of-plane stray field gradient. As shown in the newly added Fig. S22, no discernible magnetic contrast or field-dependent evolution is observed on the micrometer scale. The small blue spot is likely a defective region of the crystal serving as a reference point for scanning the same field of view. The absence of MFM signal is fully consistent with the altermagnetic nature of the SDW ground state inferred from our STM observations and DFT calculations, and further excludes the magnetic phase transition as the origin of the magnetic-field-induced sliding CDW.

Accordingly, we have added the measurement details and discussion in the supplementary information as Section F.

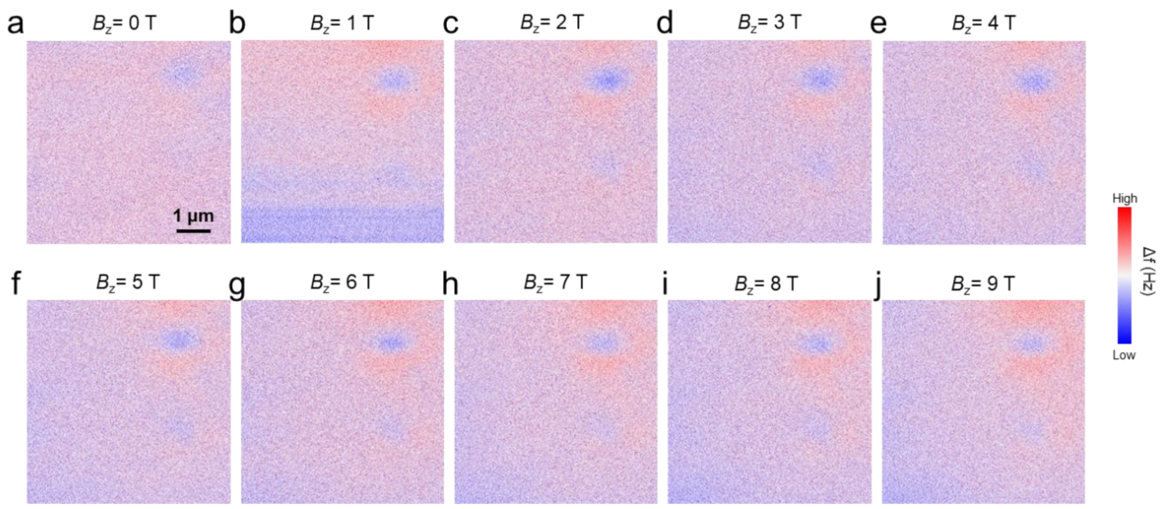

New Fig. S22. Magnetic force microscopy measurements under different magnetic fields at 50 mK. **a-j**, Spatial maps of the frequency shift (Δf) acquired at B_z of 0-9 T. No discernible field-induced evolution of the Δf signal was detected across the entire investigated field range. The defect area in the upper right (small blue region) serves as a reference point for scanning the same field of view.

Comment 4: *More cases have been reported in which the CDW responds to an external magnetic field. The relevant references are provided below.*

Xing et al. *https://doi.org/10.1038/s41586-024-07519-5*
Ichicawa et al. *https://doi.org/10.1038/s41699-025-00584-y*

We sincerely appreciate the Reviewer for pointing out the references, which are now being cited in the discussion section of the revised manuscript.

"The magnetic field–induced evolution of CDW patterns observed is highly unconventional. For instance, in AV_3Sb_5 system, the chirality of the $2a_0 \times 2a_0$ CDW order can be tuned by magnetic field[51], indicating its exotic time-reversal-breaking nature. In strained 2H-$NbSe_2$, an exotic stripe order can be induced by magnetic field[52] from $2a_0 \times 2a_0$ CDW phase. In typical systems, CDW states arise from charge modulation due to Fermi surface nesting or electron–phonon coupling and do not possess intrinsic magnetic components…"

Comment 5: *Xing et al. put forward electro-striction and piezo-magnetic responses as possible mechanisms, rather than the authors' stated interpretation. These appear to explain the experimental results more simply.*

We sincerely thank the Reviewer for pointing out the paper by Xing *et al.*[*Nature* **631**, 60–66 (2024)] and for raising the possibility of electro-striction and piezomagnetic responses as alternative mechanisms. We agree that these effects provide an elegant explanation for magnetic- and light-induced responses of CDWs in certain systems, and we carefully examined whether they could account for our observations.

Xing *et al.* seem to attribute the magnetic-field response of the CDW to a piezomagnetic effect, which manifests as a magnetic-field-induced lattice deformation. Experimentally, this mechanism is supported by a measurable change in the lattice constants, visible as a systematic shift in the Bragg peak positions in Fourier-transformed STM images under different magnetic fields.

If the magnetic-field-induced CDW sliding observed in our experiment originated from a piezomagnetic response, a similar field-dependent modification of the lattice constant would be expected. Specifically, the magnitudes of the Bragg wave vectors should change with applied magnetic field.

To directly test this possibility, we carefully compared the Bragg peak positions extracted from STM images acquired at 0 T, 4.5 T, and 9 T. The results are presented in the newly added Fig. S23. As shown in Fig. S23**c**, the Bragg peaks at different fields overlap within experimental uncertainty, indicating that the lattice constants remain unchanged across the entire magnetic-field range.

This observation demonstrates that any piezomagnetic lattice distortion, if present, is negligible in our system. Consequently, the magnetic-field-induced CDW evolution we report cannot be explained by a piezomagnetic mechanism.

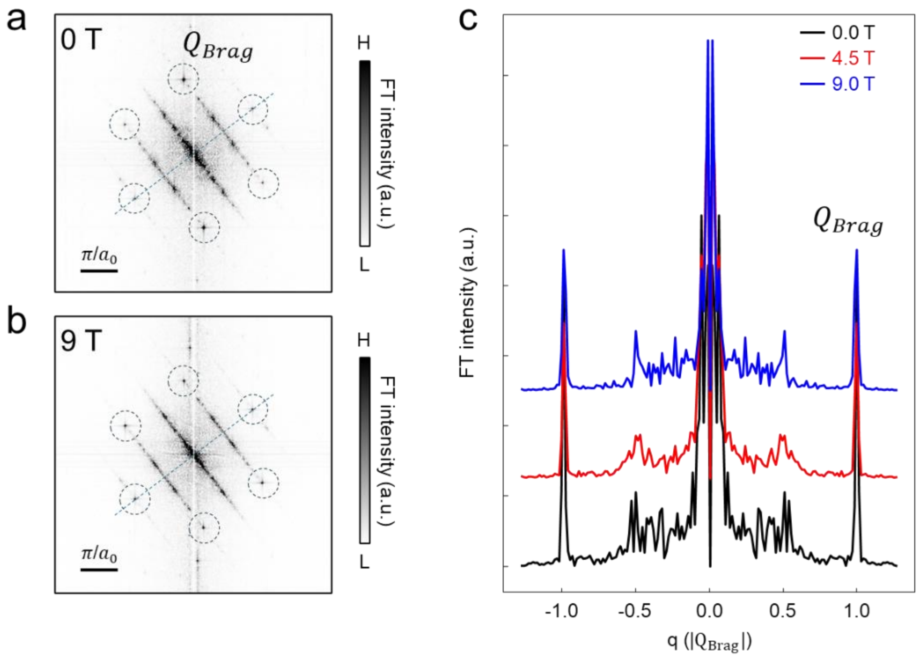

New Fig. S23. Inspection of piezo-magnetic response. a, Fourier transform (FT) of the STM topography at 0 T (same field of view as Fig. 4**a**), showing Bragg peaks. Measurement conditions: V_b=-50 mV, I=1 nA. **b**, FT of the STM topography at 9 T (same field of view), showing Bragg peaks. Measurement conditions: V_b=-50 mV, I=1 nA. **c**, Comparison of the Bragg wavevector magnitude $|Q_{Brag}|$ under different magnetic field, indicating no detectable change in lattice constant within the experimental resolution. The FT line profiles were extracted along the dashed lines marked in (**a**) and (**b**).

Instead, as also detailed in our response to reviewer 1, we may understand the spin dynamics based on a simple toy model to simulate the spin dynamics of the SDW. This model is based on one-dimensional antiferromagnetic chain with periodic boundary conditions, using the Landau-Lifshitz-Gilbert (LLG) equation. The simulation results show that the in-plane spin component S_x exhibits a cosine-wave modulation under an out-of-plane field B_z (Fig. S15**c**), whose phase displays a linear dependence on the magnetic field (Fig. S15**d**). This trend qualitatively agrees with the linear variation of the CDW stripe phase observed experimentally, further suggesting that the magnetic-field manipulation of the CDW may originate from the magnetic field-driven modulation of the underlying SDW.

We have added the calculation details in the supplementary information as Section A and new Fig. S15. We have also added the discussion of the possible piezomagnetic response in the supplementary information as Section G.

In summary, we believe we have carefully addressed both the Reviewers' comments and questions by adding new data and discussions, as well as clarifying revisions. Once again, we sincerely thank both Reviewers for their insightful, constructive and critical comments/questions, which have helped us to further strengthen the manuscript, which, we hope, now makes it suitable for publication in *Nature Communications*.

Responses to the reviewers' comments

Response to the Reviewer #1

Comment: The authors have addressed my concerns, now I recomend the manuscript for publication.

We sincerely thank the Reviewer for their positive assessment and for recommending our manuscript for publication.

Response to the Reviewer #2

Comment: The authors have generally provided appropriate responses to the issues I raised. I recommend this manuscript for publication.

We sincerely thank the Reviewer for their constructive feedback and for recommending our manuscript for publication.
